# Lipopeptide Interplay Mediates Molecular Interactions between Soil Bacilli and Pseudomonads

**DOI:** 10.1128/spectrum.02038-21

**Published:** 2021-12-08

**Authors:** Sofija Andrić, Thibault Meyer, Augustin Rigolet, Claire Prigent-Combaret, Monica Höfte, Guillaume Balleux, Sébastien Steels, Grégory Hoff, René De Mot, Andrea McCann, Edwin De Pauw, Anthony Argüelles Arias, Marc Ongena

**Affiliations:** a Microbial Processes and Interactions Laboratory, Terra Teaching and Research Centre, Gembloux Agro-Bio Tech, University of Liège, Gembloux, Belgium; b UMR Ecologie Microbienne, University of Lyon, Université Claude Bernard Lyon 1, CNRS, INRAE, VetAgro Sup, Villeurbanne, France; c Laboratory of Phytopathology, Department of Plants and Crops, Faculty of Bioscience engineering, Ghent University, Ghent, Belgium; d Centre of Microbial and Plant Genetics, Faculty of Bioscience Engineering, University of Leuven, Heverlee, Belgium; e Mass Spectrometry Laboratory, MolSys Research Unit, Department of Chemistry, University of Liège, Liège, Belgium; University of Minnesota

**Keywords:** *Bacillus velezensis*, molecular cross-talk, *Pseudomonas*, bioactive secondary metabolites, cyclic lipopeptides, microbial ecology, microbial interaction, plant growth-promoting rhizobacteria

## Abstract

Some *Bacillus* species, such as B. velezensis, are important members of the plant-associated microbiome, conferring protection against phytopathogens. However, our knowledge about multitrophic interactions determining the ecological fitness of these biocontrol bacteria in the competitive rhizosphere niche is still limited. Here, we investigated molecular mechanisms underlying interactions between *B. velezensis* and Pseudomonas as a soil-dwelling competitor. Upon their contact-independent *in vitro* confrontation, a multifaceted macroscopic outcome was observed and characterized by *Bacillus* growth inhibition, white line formation in the interaction zone, and enhanced motility. We correlated these phenotypes with the production of bioactive secondary metabolites and identified specific lipopeptides as key compounds involved in the interference interaction and motile response. *Bacillus* mobilizes its lipopeptide surfactin not only to enhance motility but also to act as a chemical trap to reduce the toxicity of lipopeptides formed by Pseudomonas. We demonstrated the relevance of these unsuspected roles of lipopeptides in the context of competitive tomato root colonization by the two bacterial genera.

**IMPORTANCE** Plant-associated Bacillus velezensis and Pseudomonas spp. represent excellent model species as strong producers of bioactive metabolites involved in phytopathogen inhibition and the elicitation of plant immunity. However, the ecological role of these metabolites during microbial interspecies interactions and the way their expression may be modulated under naturally competitive soil conditions has been poorly investigated. Through this work, we report various phenotypic outcomes from the interactions between *B. velezensis* and 10 Pseudomonas strains used as competitors and correlate them with the production of specific metabolites called lipopeptides from both species. More precisely, *Bacillus* overproduces surfactin to enhance motility, which also, by acting as a chemical trap, reduces the toxicity of other lipopeptides formed by Pseudomonas. Based on data from interspecies competition on plant roots, we assume this would allow *Bacillus* to gain fitness and persistence in its natural rhizosphere niche. The discovery of new ecological functions for *Bacillus* and Pseudomonas secondary metabolites is crucial to rationally design compatible consortia, more efficient than single-species inoculants, to promote plant health and growth by fighting economically important pathogens in sustainable agriculture.

## INTRODUCTION

Bacilli belonging to the Bacillus subtilis complex are ubiquitous members of the rhizosphere microbiome, which contains a subset of the bulk soil microbes that evolve to dwell in this compartment surrounding roots, nutrient-enriched due to continued exudation (1–3). Among these species, Bacillus velezensis is emerging as a model for plant-associated bacilli and displays strong potential as a biocontrol agent, reducing diseases caused by phytopathogens (4). *B. velezensis* is distinguished from other species of the B. subtilis group by its richness in biosynthetic gene clusters (BGCs; representing up to 13% of the whole genome) responsible for the synthesis of bioactive secondary metabolites (BSMs) (5, 6). This chemically diverse secondary metabolome includes volatiles, terpenes, ribosomally synthesized lantibiotics and bacteriocins (RiPPs), and nonribosomally (NR) synthesized metabolites, such as polyketides (PKs), dipeptides, siderophores, and cyclic lipopeptides (CLPs) (7, 8). Due to their amphiphilic and antimicrobial properties, CLPs of the surfactin, iturin, and fengycin families are clearly involved in the biocontrol activity of the producing strains via direct inhibition of phytopathogenic microbes and/or via stimulation of the plant immune system, leading to induced systemic resistance against aggressors (9–13). Moreover, these multifunctional compounds may also act as drivers of some developmental traits in multicellular communities, such as biofilm formation and motility, thereby contributing more globally to bacterial competitiveness in the rhizosphere niche.

However, our knowledge about the production and biological activities of *Bacillus* CLPs or BSMs in general relies mainly on data obtained from monocultures of the producing strains, which is far from natural conditions. Soil is indeed one of the richest ecosystems in terms of microbial diversity and abundance (14), but the scarcity of resources makes it also one of the most privileged environments for competitive interspecies interactions (1, 15). As the rhizosphere is more densely populated (15) than the bulk soil, it is assumed that microbial warfare in this habitat is even more intense. In that context, both rivalries for nutrients and interference competition are considered key factors driving microbial interactions and community assembly (16). The production and role of *Bacillus* BSMs may thus undergo anticipated changes following interaction with other species from the same niche, but this interplay remains poorly understood (17). Some previous works have illustrated how soil bacilli may adapt their behavior and protect themselves upon sensing bacterial competitors, notably by improving biofilm formation (18–25), enhancing motility (26, 27), or inducing sporulation (28, 29). Most of these reports are focused mainly on the adaptation of such developmental traits, but there is little information about *Bacillus* response at the molecular level following interspecies interactions (17).

Through this work, we wanted to further investigate the possible roles of BSMs in the molecular interactions of *B. velezensis* with other bacterial species. We selected fluorescent pseudomonads as challengers because these bacteria are also prominent among the rhizosphere microbial community (1–3, 30). Numerous species have been amply described for their biocontrol capacities, are genomically well characterized, and produce a wide array of secondary metabolites (31–33). This includes various antibiotics (e.g., phenazines, phloroglucinols, pyoluteorin, and pyrrolnitrin) but also CLPs formed by many species belonging to the P. fluorescens group. There is a huge structural diversity among Pseudomonas CLPs reported to date that are classified into 14 groups according to the length of the oligopeptide and the size of the macrolactone ring (34, 35). However, in contrast to *B. velezensis*, most plant-associated and beneficial Pseudomonas isolates only produce one type of CLP (36). Studies on interactions between *Bacillus* and specific Pseudomonas isolates that have been reported recently revealed sophisticated competition strategies between these two genera (37–41).

Here, in order to highlight the possible role of BSMs as small-size diffusible compounds, we first used contact-independent settings for investigating pairwise interaction between *B. velezensis* and 10 Pseudomonas isolates. The two bacteria initiated multifaceted interactions illustrated by *Bacillus* growth inhibition, enhanced motility, and white line formation near the *Bacillus* colony as main outcomes. We could correlate these phenotypes with BSM production and identified specific lipopeptides as the main compounds involved in the interference interaction and motile response. We also illustrated the relevance of these unsuspected roles of CLPs in the context of competitive tomato root colonization.

## RESULTS

### Diverse bioactive secondary metabolites of plant-associated Pseudomonas are involved in interaction with *Bacillus*.

**FIG 1:**
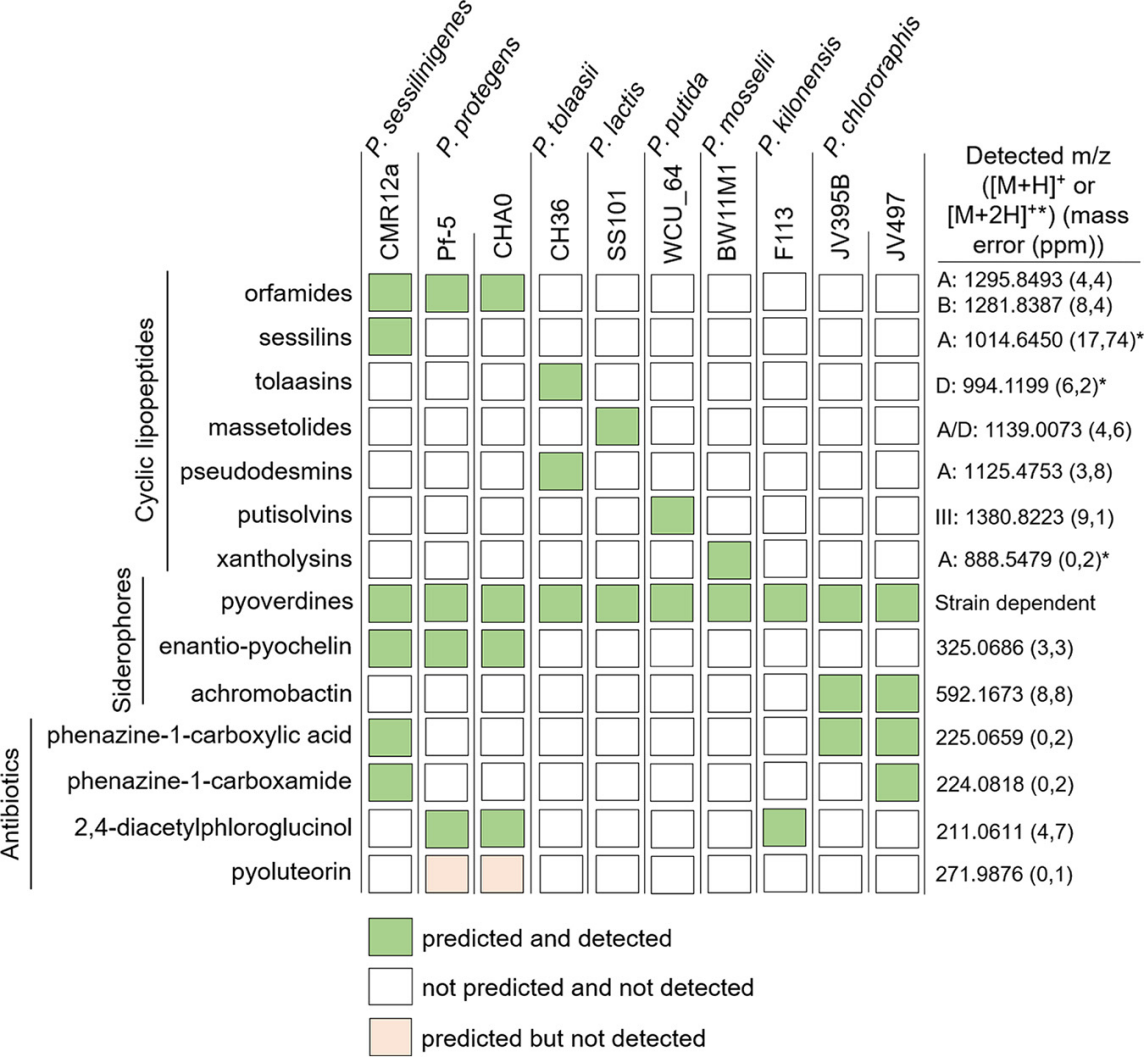
Predicted and detected bioactive secondary metabolites (BSMs) produced by the Pseudomonas strains used in this study. Metabolites with biosynthetic gene clusters (BGCs) predicted by antiSMASH 6.0 (42) and detected ([M+H]^+^ or [M+2H]^+^, the latter is indicated with an asterisk) by UPLC-qTOF MS in crude cell-free supernatant after growth in CAA medium are represented by green squares, while undetected BSMs are represented by light red squares. Detected *m*/*z* and mass errors in parts per million (based on one measurement from one strain [orfamides, CMR12a; achromobactin, JV497; phenazines, CMR12a; 2,4-diacetylphloroglucinol, Pf-5]) corresponding to the main variant(s) (indicated with the letters) detected are presented. The data are representative of the two independent repetitions.

We first evaluated the interaction outcomes following confrontation of *B. velezensis* strain GA1 with several plant-associated Pseudomonas strains belonging to different species. We used the recently sequenced (GenBank: CP046386) *B. velezensis* strain GA1 as a BSM-rich and genetically amenable isolate representative of the species. Genome mining with antiSMASH 6.0 (42) confirmed the presence of all BGCs necessary for the biosynthesis of known BSMs typically formed by this bacterium (Fig. S1A in the supplemental material). Based on the accurate mass determined via ultrahigh performance liquid chromatography-quadrupole time-of-flight mass spectrometry (UPLC-qTOF MS) (Fig. S1B and C), most of the predicted NR secondary metabolites were identified in cell-free crude supernatants obtained from the culture in a so-called exudate-mimicking (EM) medium. This medium has been used in this study mostly to grow *Bacillus* as it reflects the specific content in major carbon sources typically released by roots of *Solanaceae* plants, such as tomato and tobacco (43). Most compounds were also produced, but in smaller amounts, following growth in casamino acid (CAA) medium, commonly used for secondary metabolite production by Pseudomonas (Fig. S1B). The same approach combining genome mining (when available) and UPLC-qTOF MS was used to determine the BSMs produced by each of the 10 Pseudomonas strains grown as planktonic cells in liquid CAA (Fig. 1). All isolates produced the siderophore pyoverdine, typical of fluorescent Pseudomonas species, but some strains also synthesized a secondary siderophore such as (enantio-)pyochelin or achromobactin with a lower affinity for iron (44). The 10 Pseudomonas strains also differed in their potential to form broad-spectrum antibiotics, such as phenazine derivatives (phenazine-1-carboxamide [PCN] and phenazine-1-carboxylic acid [PCA]), 2,4-diacetylphloroglucinol (DAPG), and/or pyoluteorin (32, 36, 45). CLPs represent the most structurally diverse group of metabolites in the selected isolates (see Fig. S2 for the CLP structures). Most strains produced a single CLP belonging either to the viscosin (massetolide) (Pseudomonas lactis SS101), orfamide (Pseudomonas protegens Pf-5 and CHA0), putisolvin (Pseudomonas putida WCU_64), and xantholysin (Pseudomonas mosselii BW11M1) groups. *P. sessilinigenes* CMR12a and Pseudomonas tolaasii CH36 were the only strains coproducing two structurally distinct CLPs (sessilins and orfamides or tolaasins and pseudodesmins, respectively), while no CLPs were predicted and detected in the extracts from P. chlororaphis JV497 and JV395B or Pseudomonas kilonensis F113 (Fig. 1).

**FIG 2:**
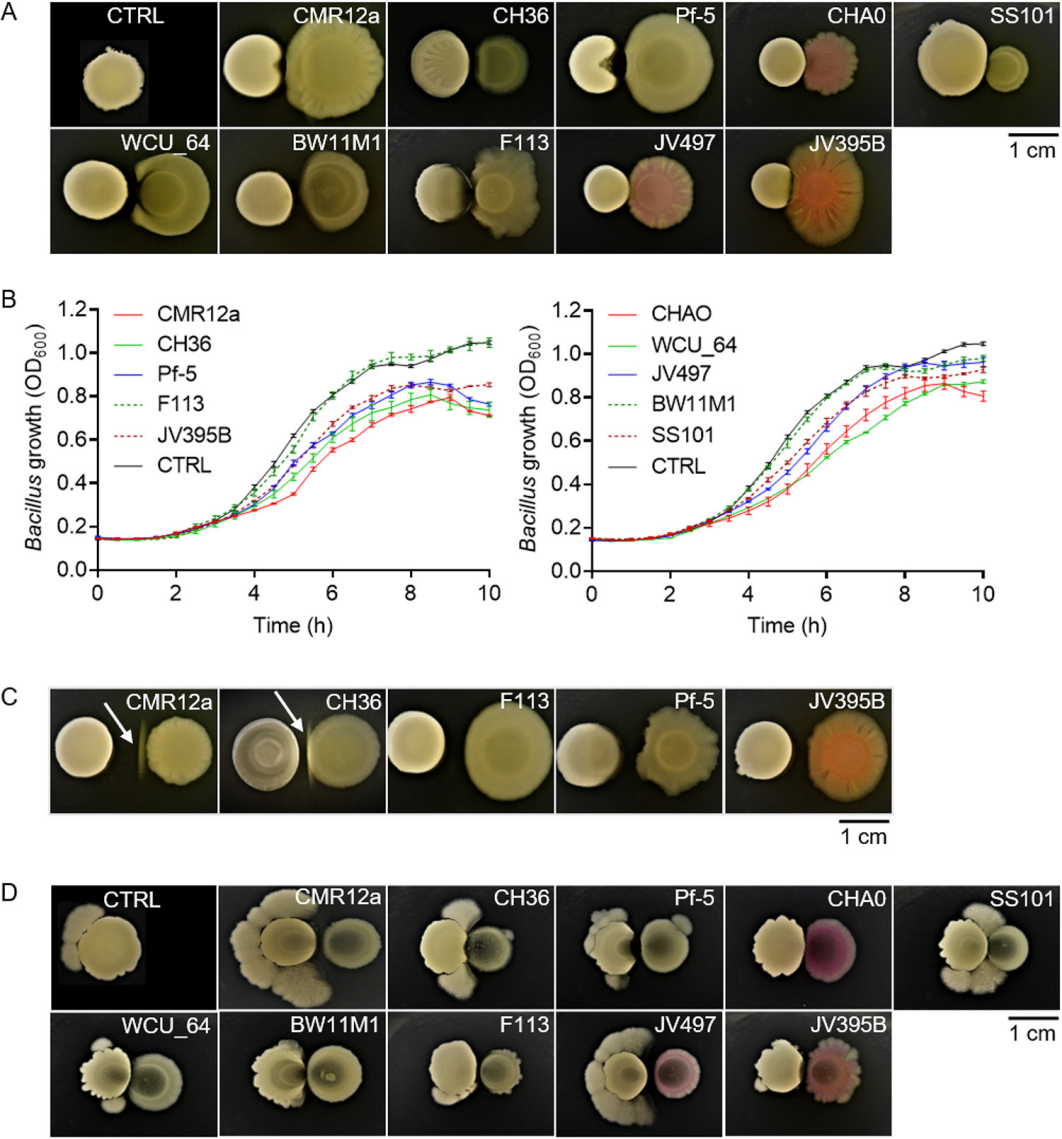
Phenotype and growth of *B. velezensis* GA1 following confrontation with different Pseudomonas strains. (A) *B. velezensis* GA1 (left colony) phenotypic response on solid CAA medium following short distance (1 mm) confrontation with different Pseudomonas strains (right colony). The control (CTRL) colony shows GA1 cultured alone. (B) GA1 growth curve in EM liquid medium supplemented with 4% (vol/vol) of different Pseudomonas cell-free supernatants. The control (CTRL) corresponds to unsupplemented GA1 culture (data represent means ± standard deviation [SD]; *n* = 9). (C) White line formation (indicated by arrow) between colonies of GA1 (left colony) and some Pseudomonas strains (right colony) on jellified CAA medium following longer distance (5 mm) confrontation. (D) GA1 (left colony) motile response on EM medium following confrontation with different Pseudomonas strains (right colony). The control (CTRL) colony shows GA1 cultured alone. Pictures in panels A, C, and D are representative of the response observed in three independent repetitions with three technical replicates (*n* = 9).

Following dual confrontation on solid CAA medium, strains JV395B, F113, Pf-5, CH36, and CMR12a displayed some inhibitory activity toward the growth of GA1 colonies due to the production of soluble metabolites diffusing in the interaction zone (Fig. 2A). This inhibitory activity was also illustrated in liquid cultures when growth reduction of GA1 planktonic cells was observed following supplementation of the medium with Pseudomonas cell-free supernatant (CFS), previously prepared from the culture in CAA medium (Fig. 2B). However, the relative inhibitory activity of the different Pseudomonas isolates varied according to the experimental conditions on plates or in liquid cultures. For instance, the addition of CFS from strains CHA0, WCU_64, JV497, and SS101 strongly reduced GA1 cell growth in liquid culture, while the corresponding isolates displayed only low inhibition of *Bacillus* colonies on solid medium (Fig. 2A and B). This may be explained by the fact that for some isolates, the antibiotic production rate is very different for cells forming biofilm-like microcolonies on plates compared to planktonic cells in liquid cultures, as already reported (32).

However, the Pseudomonas inhibitory effect was strongly reduced or disappeared between the bacteria confronted at a longer distance where, interestingly, the formation of a white line was observed between the bacterial colonies in the case of CMR12a and CH36 but not for other strains (Fig. 2C). Additional confrontation assays performed on EM medium showed enhanced motility of GA1 as another phenotype observed in response to Pseudomonas, which was the most marked following confrontation with CMR12a and JV497 (Fig. 2D).

### The interplay between CLPs drives antagonistic interactions and white line formation.

**FIG 3:**
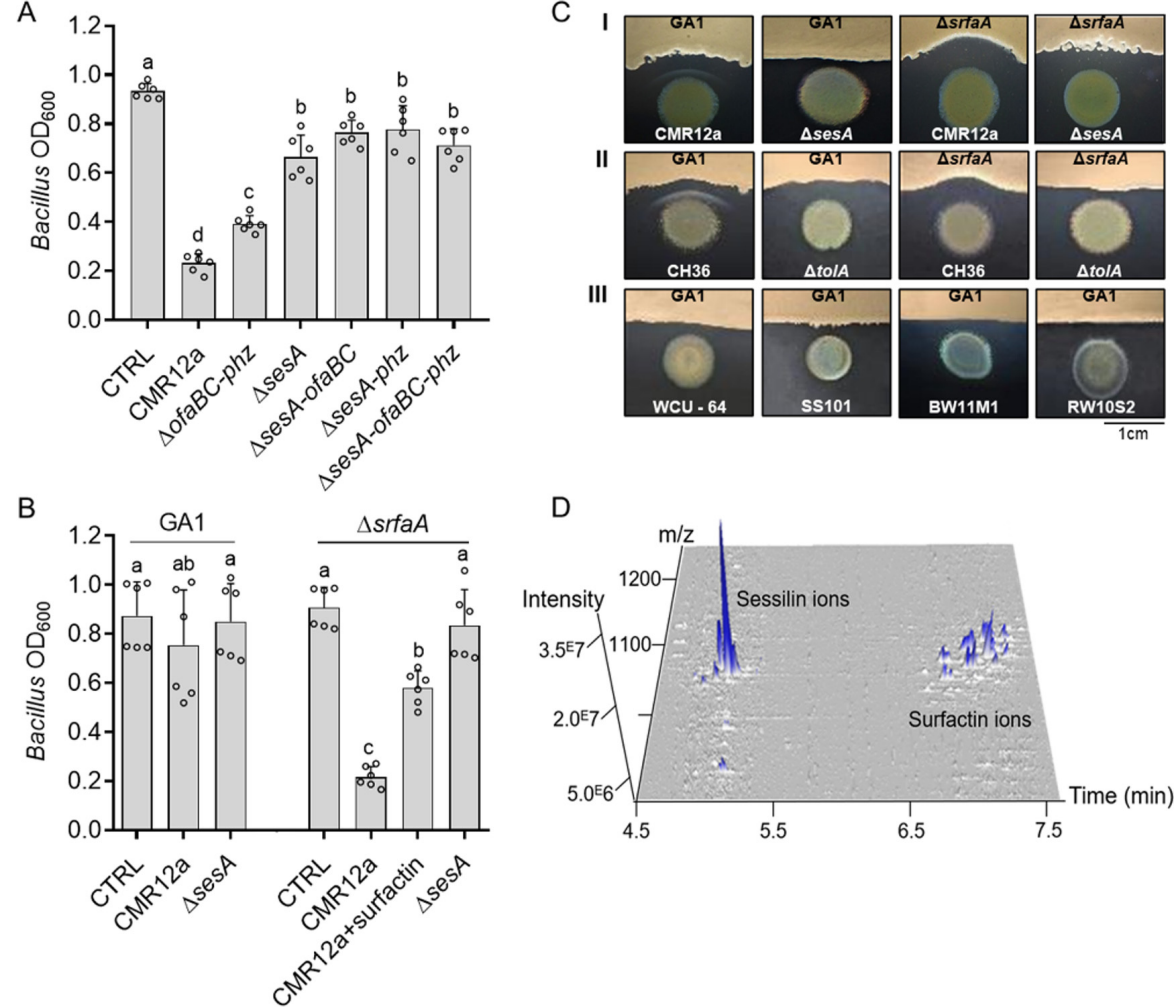
Surfactin attenuates sessilin-mediated toxicity via white line formation. (A) GA1 biomass level measured after 10 h of growth in EM liquid medium supplemented or not (CTRL) with 4% (vol/vol) of cell-free supernatants from CAA cultures of CMR12a wild type or its mutants repressed in the synthesis of orfamides and phenazines (Δ*ofaBC-phz*), sessilins (Δ*sesA*), sessilins and orfamides (Δ*sesA-ofaBC*), sessilins and phenazines (Δ*sesA-phz*), or all compounds (Δ*sesA-ofaBC-phz*) (for metabolome, see Table S1 in the supplemental material). Data show mean ± SD calculated from two independent experiments each with three culture replicates (*n* = 6) and different letters indicating statistically significant differences between the treatments (ANOVA and Tukey’s HSD tests, *α =* 0.05). (B) Growth inhibition of GA1 wild type and its Δ*srfaA* mutant repressed in surfactin synthesis after 10 h of culture and following delayed supplementation (added 6 h after incubation start) with cell-free supernatants from CMR12a wild type (alone or together with 10 µM pure surfactin as chemical complementation) and with cell-free supernatants from the sessilin mutant (Δ*sesA*). Unsupplemented cultures of GA1 were used as a control (CTRL). Experiments were replicated, and data were statistically processed as described in panel A (*n* = 6). (C) White line formation and/or *Bacillus* inhibition observed following confrontation of GA1 wild type or the surfactin mutant Δ*srfaA* with (I) CMR12a or its Δ*sesA* derivative, (II) *P. tolaasii* CH36 or its tolaasin-defective mutant Δ*tolA*, and (III) other Pseudomonas CLP producers (for metabolome, see Table S1). Pictures are representative of three independent repeats. (D) 3D representation of UPLC-MS analysis of metabolites that are present in the white line zone between GA1 and CMR12a showing the specific accumulation of sessilin and surfactin molecular ions.

Based on these data, we selected CMR12a as a challenger for further investigation of the molecular basis of the interactions because the three responses of GA1 (inhibition, white line formation, and enhanced motility) were observed following confrontation with this strain. First, we hypothesized that the inhibitory effect of CMR12a could be due to the production of CLPs and/or phenazines as main compounds with potential antimicrobial activity (Fig. 1). We, therefore, tested the effect of CFS from various CMR12a mutants impaired in the synthesis of these metabolites. As already described, the production level of the nonimpaired metabolites in each of the mutants was not affected (46). CFS of the mutant unable to produce sessilins (Δ*sesA*) displayed a significantly reduced ability to inhibit GA1 growth compared to CFS of CMR12a wild type (Fig. 3A). Moreover, a similarly reduced ability to inhibit GA1 has been observed in double or triple mutants affected both in sessilin and orfamide synthesis or in CLP and phenazine production, whereas the growth of GA1 was weakly reduced by a double mutant affected in phenazine and orfamide synthesis (Fig. 3A). Even if some minor effects of the other compounds could not be ruled out, it indicated that sessilins are the main CMR12a metabolites responsible for toxic activity toward GA1 cells. Nevertheless, we observed that the sessilin-mediated inhibitory effect was markedly reduced by delaying CMR12a CFS supplementation until 6 h of GA1 culture instead of adding it at the beginning of the incubation (Fig. 3B). This suggested that some early secreted GA1 compounds may counteract the toxic effect of sessilins. We hypothesized that surfactin can play this role as it is the first detectable BSM to accumulate in significant amounts in the medium early in the growth phase (47). We tested a surfactin-deficient mutant and observed that its growth was more strongly affected than GA1 wild type upon CMR12a CFS supplementation. Chemical complementation with purified surfactin restored growth of the Δ*srfaA* mutant to a large extent, providing further evidence for a protective role of this lipopeptide toward sessilin toxicity (Fig. 3B).

Such sessilin-dependent inhibition also occurred when bacteria were confronted on solid CAA medium favoring Pseudomonas BSM production. Under these conditions, the formation of a white precipitate in the interaction zone was observed with CMR12a wild type but not when GA1 was confronted with the Δ*sesA* mutant (Fig. 3C, panel I). UPLC-MS analysis of ethanol extracts from this white line area confirmed the presence of sessilin ions but also revealed an accumulation of surfactin from GA1 in this zone (Fig. 3D). The involvement of surfactin in precipitate formation was confirmed by the absence of this white line upon testing the Δ*srfaA* mutant in confrontation with CMR12a (Fig. 3C, panel I). Interestingly, the loss of surfactin production and white line formation was associated with a higher sensitivity of the GA1 colony to the sessilin toxin secreted by CMR12a. Altogether, these data indicate that surfactin acts as a protective agent, preventing the GA1 colony from sessilin toxicity via coaggregation into insoluble complexes.

Similar CLP-dependent antagonistic interaction and white line formation were observed following cocultivation of GA1 with *P. tolaasii* strain CH36 producing tolaasins (Fig. 3C, panel II), a CLP structurally similar to sessilins (only differing by two amino acid residues) (Fig. S2). However, this chemical interaction leading to coprecipitation is quite specific regarding the type of CLPs involved, as it was not visible following confrontation of GA1 with other Pseudomonas strains forming CLPs belonging to different structural groups that were not toxic for GA1 (Fig. 3C, panel III; see Fig. S2 for structures). White line formation and sessilin/tolaasin-dependent toxicity were also observed when other surfactin-producing *B. velezensis* strains (FZB42, S499, and QST713) were confronted with CMR12a and CH36 on solid medium and in liquid medium (Fig. S3A and B, respectively).

The chemical basis and stoichiometry of such molecular interaction between surfactin and sessilin leading to white line formation remain to be determined. However, it probably follows similar rules as observed for the association between sessilins/tolaasins and other Pseudomonas CLPs, such as white line-inducing principle (WLIP) or orfamides (35) or between CLPs and other unknown metabolites (48, 49).

### Pseudomonas triggers enhanced surfactin-mediated motility of *Bacillus*.

**FIG 4:**
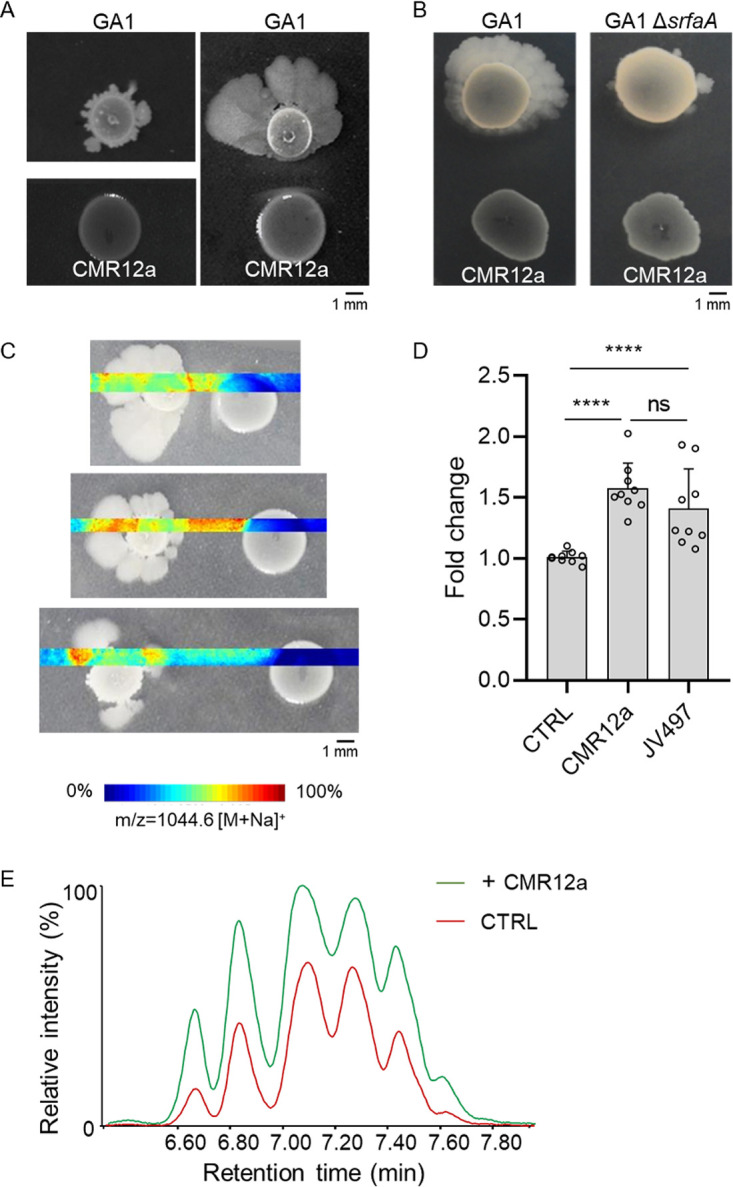
Distance- and surfactin-dependent enhanced motility of *B. velezensis* GA1 mediated by interaction with Pseudomonas. (A) GA1 motility phenotype on EM jellified medium when cultured alone (left) or in confrontation with CMR12a (1 mm) (right). (B) Motility pattern of GA1 or the Δ*srfaA* surfactin-deficient mutant in confrontation with CMR12a (5 mm). (C) MALDI FT-ICR mass spectrometry imaging (MSI) heat maps showing spatial localization and relative abundance of ions corresponding to the C_14_ surfactin homolog (most abundant) when GA1 is confronted with CMR12a at increasing distances. (D) Comparison of surfactin production (expressed in fold change) in GA1 culture supplemented with 4% (vol/vol) of CMR12a or JV497 supernatants. The unsupplemented culture was fixed at 1 (CTRL). Bars show mean ± SD (*n* = 9). Statistical comparisons between treatments were assessed with the Mann-Whitney test; ns, not significant; ****, *P < *0.0001. (E) UPLC-MS extracted ion chromatogram (EIC) illustrating the relative abundance of surfactin produced in GA1 EM medium when cultured alone (CTRL in red) or supplemented with 4% (vol/vol) CMR12a (+CMR12a in green). The different peaks correspond to the structural variants differing in fatty acid chain length.

We next wanted to better understand the observed impact of CMR12a on the motile phenotype of GA1 upon cocultivation on solid EM medium (Fig. 2D). This phenotype occurring on medium containing high agar concentrations (1.5% [mass/vol]) macroscopically resembles the sliding-type of motility (50) (Fig. 4A). This migration pattern is flagellum independent but depends on multiple factors, including the synthesis of surfactin, which reduces friction at the cell-substrate interface (50–52). We, therefore, suspected such improved motility to correlate with increased production of this lipopeptide. This hypothesis was supported by the almost full loss of migration of the Δ*srfaA* mutant under these interaction conditions (Fig. 4B). We also observed a distance-dependent effect of CMR12a on GA1 motility (Fig. 4C). The spatial mapping via matrix-assisted laser desorption/ionization-Fourier transform-ion cyclotron resonance mass spectrometry (MALDI-FT-ICR MS) imaging confirmed a higher accumulation of surfactin ions in the interaction zone and around the GA1 colony when growing at a short or intermediate distance from the Pseudomonas challenger than at the largest distance where the motile phenotype was much less visible (Fig. 4C). Stimulation of surfactin synthesis by GA1 colonies as a response to Pseudomonas perception was supported by significantly enhanced surfactin production by planktonic GA1 cells upon supplementation with CMR12a but also JV497 CFS (Fig. 4D and E). These data indicate that GA1 cells in the microcolony perceive a soluble signal diffusing from the Pseudomonas colony over a limited distance.

To examine if the compounds diffusing in the interaction zone may act as a trigger of GA1 enhanced motility, we analyzed the content of produced metabolites in the interaction zone between the confronted bacteria. The UPLC-MS analysis of ethanol extracts from the confrontation area detected the presence of CLPs (sessilins and orfamides) and phenazine antibiotics (PCN and PCA) (Fig. S4A and B). In an attempt to identify the CMR12a compound responsible for triggering GA1 motility, we analyzed the effects of CMR12a mutants specifically suppressed in the production of CLPs, phenazines, and siderophores in a confrontation assay. However, none of the mutants lost the ability to enhance GA1 motility, indicating the involvement of another CMR12a metabolite as an inducer of the GA1 response (Fig. S5).

### BSM-mediated interactions drive competitive root colonization.

To appreciate the relevance of our *in vitro* observations in a more natural context, we further evaluated whether such BSM interplay may also occur upon root cocolonization and to what extent it may impact *Bacillus* rhizosphere fitness. To that end, we compared the dynamics of GA1 populations on tomato roots when inoculated alone or following coinoculation with CMR12a and its sessilin-repressed Δ*sesA* mutant or with the JV497 strain, which also triggered GA1 motility but was not inhibitory for GA1 growth following confrontation on solid medium (Fig. 2A and D). Thus, JV497, which does not produce CLPs, and Δ*sesA* represented appropriate controls for the evaluation of CLP involvement in CMR12a effects on GA1 under *in planta* conditions.

**FIG 5:**
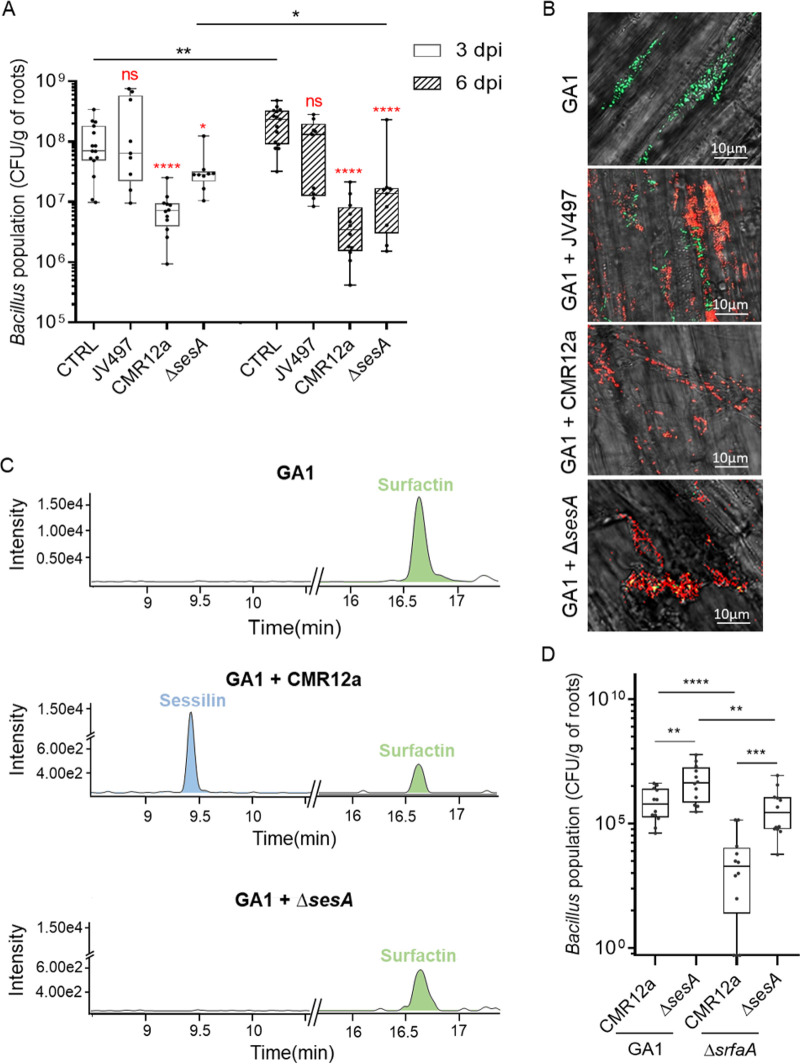
Competitive root colonization assays support the roles of BSMs in Bacillus-Pseudomonas interaction *in planta*. (A) GA1 population (CFU per gram of tomato root) recovered at 3 days (empty bars) and 6 days (hatched bars) after coinoculation (dpi) with JV497, CMR12a, and its sessilin-repressed mutant compared to GA1 inoculated alone. Box plots were generated based on data from a minimum of three biologically independent assays each involving 6 plants per treatment (*n* = 18). The whiskers extend to the minimum and maximum values, and the midline indicates the median. Statistical differences between data at the same dpi are compared to the control (CTRL) and are labeled in red, while differences between treatments at different dpi are indicated with horizontal lines and a black asterisk when significant. Statistical analyses were performed using a Mann-Whitney test; ns, no significant difference; *, *P < *0.05; **, *P < *0.01; ****, *P < *0.0001. (B) Confocal laser scanning microscopy images of tomato root colonization by GA1 (elongation root zone) at 6 dpi after monoinoculation (CTRL) or coinoculation with JV497, CMR12a, or CMR12a Δ*sesA*. GA1 tagged with GFPmut3 is depicted in green, while mCherry- or eforRed-labeled cells in red correspond to CMR12a and JV497 wild types or CMR12a Δ*sesA*, respectively. (C) UPLC-MS EIC illustrating relative production *in planta* of sessilin (blue peak) and surfactin (green peak) after 6 dpi of GA1 alone (GA1) or coinoculated with wild-type Pseudomonas (GA1 + CMR12a) or its sessilin-impaired mutant (GA1 + Δ*sesA*). (D) Cell populations recovered at 3 dpi for GA1 wild type (GA1) or the surfactin-impaired mutant (Δ*srfaA*) coinoculated with CMR12a wild type (CMR12a) or its sessilin-impaired mutant (Δ*sesA*). Box plots were generated based on data from four biologically independent assays each involving at least 4 plants per treatment (*n* = 16). The whiskers extend to the minimum and maximum values, and the midline indicates the median. Statistical differences between the treatments were calculated using a Mann-Whitney test; **, *P < *0.01; ***, *P < *0.001; ****, *P < *0.0001.

Population assessment via plate counting first revealed that following a single inoculation, the three Pseudomonas strains colonized roots more rapidly and extensively than GA1 (Fig. S6A). While Pseudomonas populations were not affected following coinoculation with GA1 (Fig. S6B to D), GA1 colonization ability was significantly reduced in the presence of CMR12a but not in the presence of JV497 after 3 days (Fig. 5A). The effect of these two wild-type Pseudomonas strains on the GA1 population was not significantly changed between the third and sixth day after coinoculation (Fig. 5A). However, GA1 colonization rate in the presence of the Δ*sesA* mutant was much less impacted (3 days postinoculation [dpi]) than coinoculation with CMR12a wild type, even if some negative effect of the Δ*sesA* mutant on GA1 colonization was observed 6 dpi (Fig. 5A). Root colonization patterns following single and dual inoculation were further investigated by confocal laser scanning microscopy (CLSM) using green fluorescent protein (GFPmut3)-tagged GA1, mCherry-tagged CMR12a or JV497, and eforRed-tagged CMR12a Δ*sesA*. When inoculated separately, GA1 was able to colonize the elongation and upper root zones and form biofilm-like multicellular communities, but no colonization of the apical meristem was observed (Fig. S7). However, Pseudomonas strains efficiently colonized the three root zones (Fig. S7). Focusing on the elongation zone, CLSM analysis of coinoculated roots (6 dpi) revealed that GA1 and JV497 or Δ*sesA* cells or microcolonies were colocalized in the same area. In contrast, GA1 was clearly excluded from CMR12a-colonized zones as no GA1 cells could be detected nearby CMR12a colonies (Fig. 5B).

Based on *in vitro* data, we hypothesized that the toxic activity of sessilins from CMR12a impacted GA1 development on roots. This is supported by the fact that this CLP was readily formed *in planta* as revealed by UPLC-MS analysis of methanolic extracts prepared from cobacterized roots and the surrounding medium (Fig. 5C). These results also confirmed the importance of interference competition due to sessilins in the early phase of colonization but also indicated that another compound or factor might be involved in *in planta* competitive interaction, excluding competition for space, as Pseudomonas spp. populations were constant during the time (Fig. S6B to D).

Besides sessilins, UPLC-MS analysis also revealed the presence of surfactin produced by GA1 during mono- or dual-colonization of tomato roots (Fig. 5C). To evaluate the importance of surfactin in GA1 colonization, a GA1 mutant unable to produce surfactin (Δ*srfaA*) was coinoculated with CMR12a wild type or the mutant unable to produce sessilins. The results showed that colonization by the Δ*srfaA* mutant was more impacted than GA1 wild type when coinoculated with CMR12a, while a significant gain in root establishment was recovered following cocolonization with the Δ*sesA* mutant (Fig. 5D). The sessilins-surfactin interplay thus also occurs *in planta*.

## DISCUSSION

Tremendous advances in analytics/genomics have vastly increased our knowledge on the diversity of bacterial BSMs, especially from species dwelling in very competitive environmental niches such as soil. It has boosted the discovery of BSMs with strong antimicrobial activity for applications in medicine and agroindustry, but most of these compounds have obvious other biological functions contributing to the persistence of the producing bacteria in natural settings. However, our vision of BSM ecological relevance is still limited, and to what extent these crucial metabolites may impact bacterial interspecies interactions is still poorly understood. Our current knowledge on the molecular interaction between soil bacilli and pseudomonads is rather limited, except the fact that the pseudomonad type VI secretion system and antibiotic 2,4-diacetylphloroglucinol may impact key developmental traits in B. subtilis, such as biofilm formation and/or sporulation rate (37, 40). Through the present work, we highlight unsuspected roles for lipopeptides, especially surfactin and sessilins, as the main BSMs driving some facets of this interaction between *B. velezensis* and Pseudomonas spp.

We illustrate that the Pseudomonas*-Bacillus* antagonistic interaction is dictated mainly by the interplay between CLPs. In contrast with short CLPs, long Pseudomonas CLPs, such as sessilins and tolaasins, retained some toxicity against *B. velezensis* as observed *in vitro*. The involvement of sessilins in the biocontrol activity of CMR12a has been well characterized and relies on antifungal activity and induced systemic resistance elicitation potential (53, 54). Here, we show that this CLP also plays a key role in bacterial interspecies interactions and competitive colonization by restricting the growth of bacterial competitors such as *Bacillus.* According to data available so far, sessilin/tolaasin-type CLPs are formed by a limited number of species, such as P. tolaasii, P. costantinii, and P. sessilinigenes isolates, as illustrated by CMR12a. This isolate has recently been taxonomically positioned as *P. sessilinigenes* (55) and has certain similarities with strains belonging to the P. protegens subgroup containing many soil Pseudomonas biocontrol strains (56–59). Considering the ever-growing number of new CLP producers discovered in recent years and the diversity/modularity of Pseudomonas NR secondary metabolites, we suspect that the potential to form CLPs from the tolaasin family could be more widespread among environmental Pseudomonas (54). In addition, other long CLPs, such as nunapeptin or corpeptin, are also formed by plant-beneficial species such as P. corrugata and P. fluorescens (60–62). They should be tested for antibacterial activity against *Bacillus* and other rhizosphere Gram-negatives in order to further support and extend the relevance of these compounds in interference competition that pseudomonads may engage in with other soil microbes. Here, we show that these CLPs also confer a significant competitive advantage to Pseudomonas for rhizosphere colonization of tomato plantlets in the presence of *Bacillus*. If this toxic activity extends to other bacteria, such sessilin-favored fitness may also apply under natural conditions.

However, this work also highlights a new role for the *Bacillus* lipopeptide surfactin in counteracting the toxicity of Pseudomonas CLPs. Our data show that surfactin acts as a protective agent that inactivates sessilins and tolaasins via coaggregation into insoluble supramolecular complexes observed as a white line on solid medium. This aggregation occurs quite specifically because this white line was not visible in confrontation with other Pseudomonas strains forming nontoxic CLPs from different structural groups. From an ecological viewpoint, the role of sessilins in white line formation for Pseudomonas species still remains unclear even if it has been proposed that sessilin production hampers the release of orfamides in the medium, which in turn reduces the swarming motility of Pseudomonas (63). Here, we provide the first example that CLP coprecipitation can be directly involved in the interference competition between two different genera. *In planta*, this new function of surfactin contributes to *Bacillus* competitiveness for root invasion. This new role as a shield to prevent sessilin-mediated toxicity has to be added to other previously reported implications of surfactin in B. subtilis interspecies interactions, such as interfering with the growth of closely related species in synergy with cannibalism toxins (64), inhibiting the development of *Streptomyces* aerial hyphae (65–68), participating in the expansion and motility of the interacting species, and also acting as a chemoattractant to Paenibacillus dendritiformis (69).

Besides this CLP interplay driving interference competition between the two species, *B. velezensis* also recruits its surfactin lipopeptide to improve multicellular mobility upon sensing Pseudomonas. This may be viewed as an escape mechanism enabling *Bacillus* cells to relocate after detecting harmful challengers. Improved motility of B. subtilis has been already described upon sensing the antibiotics chloramphenicol and linearmycins produced by competitors such as Streptomyces venezuelae and *Streptomyces* sp. Mg1, respectively (26, 27). However, no relationship was established with the enhanced production of BSMs potentially involved in the enhanced motility of B. subtilis. The competitive fitness advantage conferred by sliding mobility activation would provide an opportunity to outcompete other microorganisms for rhizosphere colonization and/or could be an escape mechanism to antimicrobials, enabling cells to relocate rapidly upon sensing competitors. As observed in this study, CMR12a mutants impaired in CLP, phenazine, and siderophore production did not lose the ability to enhance GA1 motility, indicating a possible involvement of other BSMs. Thus, the diffusible metabolite(s) from Pseudomonas that stimulate surfactin-dependent motility in nearby *Bacillus* colonies still remain to be identified. According to the principle of hormesis, antibiotics may be stimulatory metabolites at subinhibitory concentrations (11, 70), as reported for chloramphenicol produced by *S. venezuelae*, which was demonstrated to induce a mobile response in B. subtilis (26). Further in-depth investigations should be conducted to evaluate whether phenazine antibiotics, pyoverdin/pyochelin siderophores, or the other molecules may act as Pseudomonas signals, stimulating surfactin production and thus motility in *Bacillus* cells.

In conclusion, soil bacteria retain the potential to form and secrete a wide array of BSMs, but our understanding of the true ecological roles of these compounds formed under natural conditions at inhibitory or subinhibitory concentrations has just begun to be deciphered. This work points out unsuspected functions for some of these bacterial small molecules in the context of interactions between clades that are important members of the plant-associated microbiome. We postulate that the *Bacillus* metabolite response reported here largely contributes to mounting a multifaceted defensive strategy to gain fitness and persistence in its natural competitive niche. One of our challenges is to expand and integrate this knowledge to anticipate the antagonistic or mutualistic nature of the interaction to rationally design compatible consortia instead of single-species inoculants that are more efficient to promote plant growth and health toward economically important pathogens in sustainable agriculture.

## MATERIALS AND METHODS

### Bacterial strains and growth conditions.

Strains and plasmids used in this study are listed in Table S1 in the supplemental material. *B. velezensis* strains were grown at 30°C on a half-diluted recomposed exudate (EM) (43) solid medium or in liquid EM with shaking (160 rpm). Mutants of *B. velezensis* GA1 were selected on an appropriate antibiotic (chloramphenicol at 5 µg mL^−1^) in lysogeny broth (LB) (10 g L^−1^ NaCl, 5 g L^−1^ yeast extract, and 10 g L^−1^ tryptone). Pseudomonas species strains were grown on LB and CAA solid and liquid medium (10 g L^−1^ casamino acid, 0.3 g L^−1^ K_2_HPO_4_, 0.5 g L^−1^ MgSO_4_, pH 7) with shaking (160 rpm) at 30°C. When necessary, Pseudomonas spp. mutant strains were selected on appropriate antibiotics (gentamicin 20 µg mL^−1^, kanamycin 25 µg mL^−1^, or tetracycline 10 µg mL^−1^) on LB.

### Construction of deletion mutant of *B. velezensis* GA1.

A GA1 mutant unable to produce surfactin was constructed by marker replacement. Briefly, 1 kb of the upstream region of the *srfaA* gene, an antibiotic marker (chloramphenicol cassette), and the downstream region of the *srfaA* gene was PCR amplified with specific primers (Table S1). The three DNA fragments were linked by overlap PCR to obtain a DNA fragment containing the antibiotic marker flanked by the two homologous recombination regions. The latter fragment was introduced into *B. velezensis* GA1 by natural competence induced by nitrogen limitation (71). A homologous recombination event was selected by chloramphenicol resistance on LB medium. The *srfaA* gene deletion was confirmed by PCR analysis with the corresponding UpF (TCAGCAAAACTGCGTGGTAG) and DwR (AAGAAATGATCATAAATACC) specific primers and by the loss of surfactin production.

Transformation of the GA1 strain was performed following the protocol previously described (71) with some modifications. One fresh GA1 colony was inoculated into LB liquid medium at 37°C (160 rpm) until reaching an optical density at 600 nm (OD_600_) of 1.0. Afterward, cells were washed one time with peptone water and one time with a modified Spizizen minimal salt liquid medium (MMG) (19 g L^−1^ K_2_HPO_4_ anhydrous, 6 g L^−1^ KH_2_PO_4_, 1 g L^−1^ Na_3_ citrate anhydrous, 0.2 g L^−1^ MgSO_4_ 7H_2_O, 2 g L^−1^ Na_2_SO_4_, 50 µM FeCl_3_ [sterilized by filtration at 0.22 µm], 2 µM MnSO_4_, 8 g L^−1^ glucose, 2 g L^−1^ l-glutamic acid, pH 7.0), and 1 µg of DNA recombinant fragment was added to the GA1 cell suspension adjusted to an OD_600_ of 0.01 in MMG liquid medium. One day after incubation at 37°C with shaking at 165 rpm, bacteria were spread on LB plates supplemented with the chloramphenicol (5 µg mL^−1^) antibiotic to select positive colonies.

To follow *B. velezensis* GA1 colonization of roots, we designed an integrative vector region containing the *gfp* gene under the control of the *Veg* promoter (constitutive expression), which was further inserted into the GA1 chromosome by a double-crossover event at the *amyE* locus following the already described protocol (72). The tagged cells were selected on solid LB containing 5 mg mL^−1^ chloramphenicol at 30°C.

### Construction of deletion mutants of *P. sessilinigenes* CMR12a.

Enantio-pyochelin and pyoverdine mutants of CMR12a were constructed using the I-SceI system and the pEMG suicide vector (73, 74). Briefly, the upstream and downstream regions flanking the *pchA* (*C4K39_5481*) or the *pvdI* (*C4K39_6027*) genes were PCR amplified (primers listed in Table S2), linked via overlap PCR, and inserted into the pEMG vector. The resulting plasmid (Table S1) was integrated by conjugation into the CMR12a chromosome via homologous recombination. Kanamycin-resistant (25 µg mL^−1^) cells were selected on King B (KB) agar plates and transformed by electroporation with the pSW-2 plasmid (harboring the I-SceI system). Gentamycin-resistant (20 µg mL^−1^) colonies on agar plates were transferred to King B medium with and without kanamycin to verify the loss of antibiotic (kanamycin) resistance. Pseudomonas mutants were identified by PCR with the corresponding UpF (GGCATTCTTGACCGGTCGTC for *pvdI* and GACCAACTGCCGGCGGAT for *pchA*) and DwR (GGATCGAGCTGCCAAAGGAA for *pvdI* and GCGGACTGATTTCCTCGGTA for *pchA*) specific primers and via the loss of enantio-pyochelin and/or pyoverdine production.

### Construction of eforRed-tagged *P. sessilinigenes* CMR12a *ΔsesA* mutant.

To monitor *P. sessilinigenes* CMR12a *ΔsesA* mutant colonization of roots, we introduced the pME6010-*eforRed* plasmid by electroporation into *P. sessilinigenes* CMR12a Δ*sesA* (Table S1). The *eforRed* gene was under the control of the PJ23101 promoter, leading to its constitutive expression. Tetracycline-resistant (10 µg/mL) colonies containing the pME6010-*eforRed* plasmid were selected on KB agar plates after 24 h of incubation at 28°C. The presence of the plasmid in *P. sessilinigenes* CMR12a *ΔsesA* was visualized by the production of red-colored colonies due to the production of the eforRed proteins.

### Pseudomonas spp. metabolite production on solid medium.

To analyze metabolites produced by Pseudomonas spp. following confrontation with GA1 on solid medium, an area of agar (0.7 cm × 2.5 cm) near the colony was cut and transferred to Eppendorf tubes. The mass of the agar plug was recorded, and 75% acetonitrile was added in proportion to the mass of the agar plug (50 to 50 [mass/vol]). The metabolites were extracted under continuous rotation shaking at room temperature (22°C) for 30 min. The samples were centrifuged for 2 min at 10,000 rpm, and the supernatant was run through a 0.22-µm filter before UPLC-MS analysis. Data were collected from two biological repetitions.

The procedure for sample preparation and analysis of BSM production *in planta* are presented in the section Bacterial root colonization below.

### Confrontation, white line formation, and motility test.

For confrontation assays on agar plates, *B. velezensis* and Pseudomonas species strains were grown overnight in EM and CAA liquid media, respectively. After bacterial washing in peptone water and adjustment of the OD_600_ to 0.1, 5 µL of bacterial suspension was applied at a 1-mm distance onto CAA medium to observe white line formation and inhibition. For the motility assay, bacterial suspension was applied at a 1-mm, 5-mm, and 7.5-mm distance onto an EM agar plate. Plates were incubated at 30°C, and images were taken after 24 h. Photographs were captured using a CoolPix camera (NIKKOR ×60 wide optical zoom extra-low dispersion vibration reduction [EDVR] 4.3 to 258 mm 1:33 to 6.5). Three technical repetitions within each of three biological repetitions were performed for all tested strains.

### Pseudomonas spp. cell-free supernatant extraction and metabolite production in liquid medium.

To analyze BSMs produced by Pseudomonas spp. and to test the effect of Pseudomonas spp. on *Bacillus* in a liquid medium, Pseudomonas spp. cell-free supernatants were first prepared. Pseudomonas species strains were grown overnight on LB solid medium at 30°C. After washing in peptone water, the cell suspension was adjusted to an OD_600_ of 0.05 by resuspension in 100 mL of CAA or EM. Cultures were shaken at 120 rpm at 30°C for 48 h and then centrifuged at 5,000 rpm at room temperature (22°C) for 20 min. The supernatant was filter-sterilized (0.22-µm pore size filters) and stored at −20°C until further utilization and UPLC-qTOF MS analysis.

### *B. velezensis* and Pseudomonas spp. interaction in liquid medium.

To study the effect of Pseudomonas spp. on the growth of *B. velezensis* strains, the continuous growth kinetics of *B. velezensis* were followed. First, *B. velezensis* strains were grown overnight on LB solid medium at 30°C. After washing in peptone water and adjusting to an OD_600_ of 0.1, the bacterial suspension was transferred to microtiter 96-well plates, and 4% (vol/vol) Pseudomonas spp. supernatant was added, with s final volume of 200 µL per well. Control samples remained unsupplemented. The growth kinetics of *B. velezensis* (OD_600_) strains were followed every 30 min during 24 h with a Spectramax (Molecular Devices, Wokingham, UK), with continuous shaking at 30°C.

To observe the effect of CMR12a on surfactin production by GA1 in a liquid medium, GA1 cells from preculture were washed and resuspended in 2 mL of EM liquid medium to a final OD_600_ of 0.1 and placed into a 24-well plate. The bacterial culture was supplemented with 4% (vol/vol) of CMR12a cell-free supernatant, while the control remained unsupplemented. GA1 liquid cultures were shaken in an incubator at 300 rpm at 30°C for 24 h. Afterward, the *Bacillus* supernatants were sampled at 24 h and centrifuged at 5,000 rpm at room temperature (approximately 22°C) for 10 min to extract supernatants, and the cells were collected. Further, cell-free supernatants were filtered (0.22 µm) and used for analytical analysis of surfactin production.

### Analysis of BSMs produced by Pseudomonas spp. and *B. velezensis* GA1.

The cell-free supernatants of Pseudomonas spp. and GA1, prepared as previously described, were analyzed by UPLC-MS and UPLC-qTOF MS. Pseudomonas spp. metabolites were identified using an Agilent 1290 Infinity II coupled with a diode array detector and mass detector (Jet Stream ESI-Q-TOF 6530) in positive mode with the parameter set up as follows: capillary voltage of 3.5 kV, nebulizer pressure of 35 lb/in^2^, drying gas of 8 L min^−1^, drying gas temperature of 300°C, flow rate of sheath gas of 11 L min^−1^, sheath gas temperature of 350°C, fragmentor voltage of 175 V, skimmer voltage of 65 V, and octopole radiofrequency of 750 V. Accurate mass spectra were recorded in the *m*/*z* range of 100 to 1,700. A C_18_ Acquity UPLC ethylene bridged hybrid (BEH) column (2.1 mm × 50 mm × 1.7 μm; Waters, Milford, MA, USA) was used at a flow rate of 0.6 mL min^−1^ and a temperature of 40°C. The injection volume was 5 μL, and the diode array detector scanned a wavelength spectrum between 190 and 600 nm. A gradient of acidified water (0.1% formic acid) (solvent A) and acidified acetonitrile (0.1% formic acid) (solvent B) was used as mobile phase with a constant flow rate at 0.6 mL min^−1^ starting at 10% B and rising to 100% B in 20 min. Solvent B was kept at 100% for 4 min before going back to the initial ratio. MassHunter Workstation v10.0 and ChemStation software were used for data collection and analysis. Surfactin quantification was performed by using UPLC-MS with a UPLC (Acquity H-class, Waters) coupled to a single quadrupole mass spectrometer (SQD mass analyzer, Waters) using a C_18_ column (Acquity UPLC BEH C_18_ 2.1 mm × 50 mm, 1.7 µm). Elution was performed at 40°C with a constant flow rate of 0.6 mL min^−1^ using a gradient of acetonitrile (solvent B) and water (solvent A), both acidified with 0.1% formic acid as follows: 2 min at 15% B followed by a gradient from 15% to 95% during 5 min and maintained at 95% up to 9.5 min before going back to initial conditions at 10 min during 2 min before the next injection. Compounds were detected in both electrospray positive and negative ion mode by setting SQD parameters as follows: cone voltage of 60 V, source temperature of 130°C, desolvation temperature of 400°C, and nitrogen flow of 1,000 L h^−1^ with an *m*/*z* mass range from 300 to 2,048. MassLynx software v4.1 software was used for data collection and analysis. Three-dimensional (3D) chromatograms were generated using the open-source software MzMine 2 (75).

### MALDI-FT-ICR MS imaging.

Mass spectrometry images were obtained as recently described (76) using an FT-ICR mass spectrometer (SolariX XR 9.4T, Bruker Daltonics, Bremen, Germany) mass calibrated from 200 *m*/*z* to 2,300 *m*/*z* to reach a mass accuracy of 0.5 ppm. A region of interest from agar microbial colonies was directly collected from the petri dish and transferred onto an indium tin oxide (ITO) glass slide (Bruker, Bremen, Germany) previously covered with double-sided conductive carbon tape. The samples were dried under vacuum and covered with an α-cyano-4-hydroxycinnamic acid matrix solution at 5 mg/mL (70:30 acetonitrile:water [vol/vol]). In total, 60 layers of α-cyano-4-hydroxycinnamic acid matrix were sprayed using a SunCollect instrument (SunChrom, Friedrichsdorf, Germany). FlexImaging 5.0 (Bruker Daltonics, Bremen, Germany) software was used for MALDI-FT-ICR MS imaging acquisition, with a pixel step size for the surface raster set to 100 μm.

### Bacterial root colonization.

Root colonization patterns of bacteria were performed on tomato plants under gnotobiotic conditions. Tomato seeds were primarily sterilized in 70% ethanol (vol/vol) by gently shaking for 2 min. Further, ethanol was removed, and seeds were added to the 50 mL of sterilization solution (4.5 mL of bleach containing 9.5% of active chlorine, 0.01 g of Tween 80, and 45.5 mL of sterile water) and gently shaken for 10 min. Seeds were thereafter washed 10 times with water to eliminate sterilization solution residues. Sterilized seeds were placed on square petri dishes (5 seeds per plate) containing Hoagland solid medium (14 g L^−1^ agar, 5 mL of stock 1 [EDTA 5.20 mg L^−1^; FeSO_4_·7H_2_O 3.90 mg L^−1^; H_3_BO_3_ 1.40 mg L^−1^; MgSO_4_·7H_2_O 513 mg L^−1^; MnCl_2_·4H_2_O 0.90 mg L^−1^; ZnSO_4_·7H_2_O 0.10 mg L^−1^; CuSO_4_·5H_2_O 0.05 mg L^−1^; 1 mL in 50 mL stock 1, NaMoO_4_·2H_2_O 0.02 mg L^−1^ 1 mL in 50 mL stock 1], 5 mL of stock 2 [KH_2_PO_4_ 170 mg L^−1^], 5 mL of stock 3 [KNO_3_ 316 mg L^−1^, Ca(NO_3_)_2_·4H_2_O mg L^−1^], pH 6.5) and were placed in the dark to germinate for 3 days. Further, 15 germinated seeds were inoculated with 2 µL of a 6.5 × 10^8^ CFU mL^−1^ suspension (i.e., 1.3 × 10^6^ CFU/plant) of GA1, a 1.1 × 10^6^ CFU/mL suspension (i.e., 2.2 × 10^3^ CFU/plant) of JV497, a 2.2 × 10^5^ CFU/mL suspension (i.e., 4.5 × 10^2^ CFU/plant) of CMR12a, or a 1.1 × 10^5^ CFU/mL suspension (i.e., 2.2 × 10^2^ CFU/plant) of CMR12a *ΔsesA*. These inocula were prepared from overnight cultures previously washed three times in peptone water. The appropriate strains (control) or a mix of GA1 and Pseudomonas cells (coinoculation) were applied onto germinated seeds, while the same volume of sterile water solution was added for uninoculated controls. The plants were grown at 22°C under a 16-h day/8-h night cycle and 75% humidity for 6 days in the growth chamber.

For observing the effect of surfactin on GA1 root colonization following (co)inoculation of the tomato roots, slight changes in bacterial inoculation were implemented. For this purpose, 10 tomato seeds were inoculated with 2 µL of a 1.2 × 10^8^ CFU mL^−1^ suspension of GA1 (i.e., 2.4 × 10^5^ CFU/plant), a 1.1 × 10^8^ CFU/mL suspension of GA1 *ΔsrfaA* (i.e., 2.3 × 10^5^ CFU/plant), a 2.2 × 10^5^ CFU/mL suspension (i.e., 4.4 × 10^2^ CFU/plant) of CMR12a, and a 1.1 × 10^5^ CFU/mL suspension (i.e., 2.2 × 10^2^ CFU/plant) of CMR12a *ΔsesA* (control) or with a mix of *Bacillus* and Pseudomonas spp. cells (coinoculation). Plants were further grown at 22°C under a 16-h night/8-h day cycle with constant light for 3 or 6 days. After the incubation period, to determine bacterial colonization levels, bacteria from roots of six plants per condition were detached from roots and processed as explained below.

For the analysis of BSM production *in planta*, a rectangular piece of agar (1 cm width × 2.5 cm length × 0.7 cm height) surrounding the colonized root starting from the inoculation point was sampled. BSMs were extracted for 15 min with 1.5 mL of acetonitrile (85% [vol/vol]). After centrifugation for 5 min at 4,000 rpm, the supernatant was recovered for UPLC-MS analysis as previously described.

### Bacterial CFU counting.

To quantify bacterial colonization of tomato roots, 3 and 6 days after (co)inoculation, bacteria from the roots of six plants, divided into 3 samples (each containing two plants per condition), were detached from roots by vortexing for 5 min in 15-mL Falcon tubes containing 6 mL of peptone water solution supplemented with 0.1% (vol/vol) Tween 80 and 8 glassy beads. Serial dilutions were prepared, and 200 µL of each were plated onto LB medium using plating beads. Plates were incubated for 24 h at different temperatures to favor the growth of one of the interacting bacteria. Pseudomonas spp. were incubated at 28°C and GA1 at 42°C. The colonies were counted by using Scan 1200 Interscience (version 8.0.4.0). Colonization results were log transformed and statistically analyzed. At least three independent assays were performed with 3 technical repetitions (three samples each containing two plants) each for *in planta* assays.

### Confocal laser scanning microscopy analysis of bacterial root colonization.

For CLSM, samples of 1 to 2 cm in length were cut from the apical root, hair root, and mature root zones (i.e., root zone between 1 cm below the seed) and mounted in Aqua-Poly/Mount (Polysciences, Eppelheim, Germany). For analysis of green and red fluorescence emitted by bacteria, a Confocal Zeiss LSM 800 microscope (Carl Zeiss, Le Pecq, France) equipped with argon-krypton and He-Ne lasers was used by setting the excitation at 548 nm and emission between 570 and 646 nm for mCherry, the excitation at 548 nm and emission between 596 and 646 nm for eforRed, and the excitation at 488 nm and emission between 504 and 555 nm for GFPmut3. Images were processed with the LSM software, Zen 3.3 blue edition (Carl Zeiss). Five root systems were analyzed per treatment, and 3 images per root system were taken. Representative photos for each condition are presented in the figures.

### Statistical analysis.

Statistical analyses were performed using GraphPad Prism 8 software with a Student’s paired *t* test or Mann-Whitney test. For multiple comparisons, one-way analysis of variance (ANOVA) and Tukey’s honestly significant difference (HSD) tests were performed, and the groups with different letters differed significantly from each other at an *α* of 0.05. The RStudio 1.1.423 statistical software environment (R language version 4.03) was used for this purpose (77).

### Data availability.

The mass spectrometry data related to this study are publicly available and deposited in the repository MassIVE under the following digital object identifiers: https://doi.org/doi:10.25345/C5J56G, https://doi.org/doi:10.25345/C5NZ9G, https://doi.org/doi:10.25345/C5SP0J, https://doi.org/doi:10.25345/C5XG3V, https://doi.org/doi:10.25345/C52564, https://doi.org/doi:10.25345/C55Z8R and https://doi.org/doi:10.25345/C59P1K. Further data supporting the findings of this work are available in the supplemental material files. Additional data related to this paper may be requested from the authors.
