# Supplementary material for: Lipopeptide Interplay Mediates Molecular Interactions between Soil Bacilli and Pseudomonads
Source: Microbiol Spectr. 2021 Dec 8;9(3):e02038-21. doi: 10.1128/spectrum.02038-21 (PMC8653830; doi:10.1128/spectrum.02038-21)
Supplement: SUPPLEMENTAL FILE 1 — Supplemental material. Download SPECTRUM02038-21_Supp_1_seq5.pdf, PDF file, 0.9 MB [file spectrum02038-21_supp_1_seq5.pdf]

Table S1. Strains and plasmids used in this study.

	Relevant genotype, description (Genome bank number when sequenced)	References or sources
Bacillus velezensis		
GA1	Wild type (CP046386)	(1)
GA1 Δ*amyE*::cat P_{veg}-*gfpmut3* (called GA1 GFP)	GA1 disrupted of *amyE* gene; Cm^R and harbouring a constitutive transcriptional fusion (P_{veg}-*gfpmut3*).	This study
GA1 Δ*srfaA*::*cat*	GA1 deleted of *srfaA* gene; Cm^R; unable to produce surfactins	This study
S499	Wild type (NZ_CP014700)	(2)
FZB42	Wild type (NC_009725)	(3)
QST713	Wild type (NZ_CP025079)	(4)
Pseudomonas sessilinigenes		
CMR12a	Wild type (NZ_CP027706)	(5) (6)
Δ*sesA*	CMR12a disrupted of *sesA* gene; Gm^R; unable to produce sessilins	(7)
Δ*ofaBC*	CMR12a deleted of *ofaB* and *ofaC* genes; Gm^R; unable to produce orfamides	(8)
Δ*phz*	CMR12a deleted of phenazine biosynthesis operon; unable to produce phenazines	(7)
Δ*sesA-ofaBC*	CMR12a disrupted of *sesA* gene and deleted *ofaB* and *ofaC* genes; Gm^R; unable to produce sessilins and orfamides	(8)
Δ*sesA-phz*	CMR12a disrupted of *sesA* gene and deleted phenazines biosynthesis operons; Gm^R; unable to produce sessilins and phenazines	(7)
Δ*ofaAC-phz*	CMR12a deleted of *ofaB* and *ofaC* genes and phenazines biosynthesis operons; unable to produce orfamides and phenazines	(8)
Δ*sesA-ofaBC-phz*	CMR12a disrupted of *sesA* gene and deleted *ofaB* and *ofaC* genes and phenazine biosynthesis operons; Gm^R; unable to produce sessilins, orfamides and phenazines	(8)
ΔpchA	CMR12a deleted of *pchA* gene; unable to produce enantio-pyochelin	This study
ΔpvdI	CMR12a deleted of *pvdI* gene; unable to produce pyoverdine	This study
ΔpvdI-pchA	CMR12a deleted of *pvdI* and *pchA* genes; unable to produce pyoverdine and enantio-pyochelin	This study
Pseudomonas protegens		
Pf-5	Wild type; orfamide producer (NC_004129)	(9)
CHA0	*mCherry* tagged derivative of CHA0; orfamide producer (NC_021237)	(10)
Pseudomonas chlororaphis		
JV497	*mCherry* tagged derivative of JV497; non-CLPs producer (NZ_VWPC00000000)	(11)
JV395B	*mCherry* tagged derivative of JV395B; non-CLPs producer (NZ_VWPB00000000)	(11)
Pseudomonas wayambapalatensis		
RW10S2	Wild type; WLIP producer	(12)
Pseudomonas mosselii		
BW11M1	Wild type; xantholysins producer	(13)
Pseudomonas putida		
WCU_64	Wild type; putisolvins producer	(14)
Pseudomonas lactis		
SS101	Wild type; massetolides producer (NZ_CM001513)	(15)
Pseudomonas kilonensis		
F113	Wild type; viscosines producer (NC_016830)	(16)
Pseudomonas tolaasii		
CH36	Wild type; tolaasins and pseudodesmins producer	(12)
CH36Δ*tolA*	CH36 disrupted of *tolA* gene; Gm^R; unable to produce tolaasins	(12)
E. coli		
DH5α*λpir*	*supE44, ΔlacU169 (ΦlacZΔM15), recA1, endA1, hsdR17, thi-1, gyrA96, relA1, λpir*	(17)
DH5α p497	Helper strain harboring p497 plasmid	C. Keel laboratory
**Plasmids**		
pGEM-T	pGEM-T Easy derivative harboring the recombinant region *amyE*::*cat* P_{veg}-*gfpmut3* ; Cm^R	(18)
pOT1eM	pOT1e derivative harboring *Ptac-m-cherry* inserted in ClaI-SalI site ; Gm^R	(19)

pME6010-*eforRed*	pME6010 derivative harboring *PJ23101-eforRed* inserted in XhoI site; TetR	(T. Meyer, unpublished data)
pEMG	pSEVA212S; oriR6K, *lacZα* with two flanking I-*Sce*I sites; Km^r, Ap^r	(20)
pEMG-*pchA*	Suicide plasmid used for the deletion of *pchA*	This study
pEMG-*pvdI*	Suicide plasmid used for the deletion of *pvdI*	This study
pSW-2	oriRK2, *xylS*, P_m::*I-sceI*; Gm^R	(20)

Table S2. Primers used in this study.

	Primer Name	Primer sequence (5'->3')	Targeted genes
**Deletion mutant**			
***B. velezensis* GA1**			
	UpsrfaAF	TCAGCAAAACTGCGTGGTAG	*srfaA*
	UpsrfaAR	CCAATTTTCGAATTCTTTTACCGCGATAAAAAGTTATTTCCATATGTGTGC	
	DwsrfAF	CAGCTCCAGATCCTCTACGCCGGACACGCTTTATATCGTGCCGAA	
	DwsrfAR	AAGAAATGATCATAAATACC	
***P. sessilinigenes* CMR12a**			
	UppvdIF	GGCATTCTTGACCGGTCGTC	*pvdI*
	UppvdIR	GTGTTGTCCATTACACAGCCTCCATTGCATTCATCGGGAGTCATCC	
	DwpvdIF	ATGGAGGCTGTGTAATGGACAACA	
	DwpvdIR	TGTAGCGGTGTAGCAGAG	
	pvdICheckF	CCTGCTGCTGGAAGGATTGA	
	pvdICheckR	GGATCGAGCTGCCAAAGGAA	
	UppchAF	GACCAACTGCCGGCGGAT	*pchA*
	UppchAR	CCTTCAGCGATCGGCCGGTGCATCACATCTTGCGCTCCTTGCTCC	
	DwpchAF	TGATGCACCGGCCGATC	
	DwpchAR	GTGGTGAAGCTTTCCATGCC	
	pchACheckF	TCATCCACTGGAACATCGCC	
	pchACheckR	GCGGACTGATTTCCTCGGTA	
**Antibiotic marker**			
	CatF	CGCGGTAAAAGAATTCGAAAA	Chloramphenicol marker
	CatR	GTCCGGCGTAGAGGATCTG	
	PhleoF	GTCATAGCTGTTTCCTGCCAAAAGGGGGTTTCATTTT	Phleomycin marker
	PhleoR	ACTGGCCGTCGTTTTACTCCAATAAATGCGACACCAA	
	KanF	GTCATAGCTGTTTCCTGCTTAGCTCCTGAAAATCTCGG	Kanamycin marker
	KanR	ACTGGCCGTCGTTTTACCTGATAATTACTAATACTAGGAGAAG	
	nptIIF	GAGGATCGTTTCGCATGATT	Kanamycin marker for *Pseudomonas*
	nptIIR	CGCTCAGAAGAACTCGTCAA	
	psw-F	GGACGCTTCGCTGAAAACTA	pSW-II insertion
	psw-R	AACGTCGTGACTGGGAAAAC	

A

BSM predicted	Category	Biosynthetic gene cluster(s)
Surfactins	NRPs/CLPs	*srfaABCD*
Fengycins	NRPs/CLPs	*fenABCDE*
Iturins	NRPs/CLPs	*ituDABC*
Macrolactins	NRPs/PKs	*mlnABCDEFGHI*
Bacillaene	NRPs/PKs	*baeBCDEGHIJLMNRS*
Dihydrobacillaene	NRPs/PKs	*baeBCDEGHIJLMNRS*
Difficidin	NRPs/PKs	*dfnABDEFGHIJKLMNO*
Oxydifficidin	NRPs/PKs	*dfnABDEFGHIJKLMNO*
Bacillibactin	NRPs/ Siderophore	*dhbACEBF*
Bacilysin	Dipeptide	*bacABC*
Amylocyclicin	RiPP	*acnBACDEF*
Amylolysin	RiPP	*amlFEKRAMT*

C

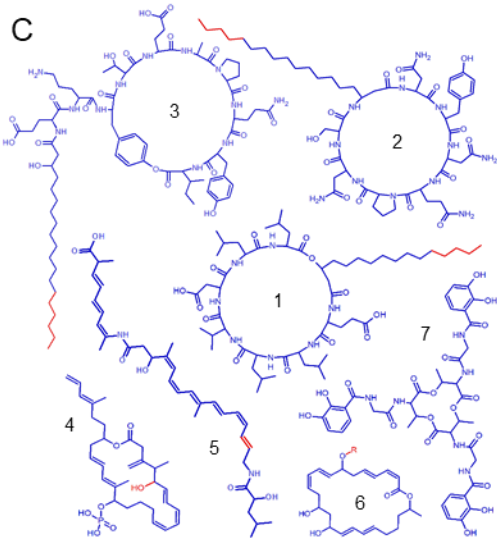

B

BSM	Main described activities	Raw formula of variants	Detected m/z [M+H]+ (mass error (ppm))	EM	CAA
Surfactins[1]	Induced systemic resistance<br><br>Motility	**C12** : $C_{50}H_{87}N_7O_{13}$<br>**C13** : $C_{51}H_{89}N_7O_{13}$<br>**C14** : $C_{52}H_{91}N_7O_{13}$<br>**C15** : $C_{53}H_{93}N_7O_{13}$<br>**C16** : $C_{54}H_{95}N_7O_{13}$	994.6420 (1,5)<br>1008.6582 (0,9)<br>1022.6743 (0,5)<br>1036.6904 (4,8)<br>1050.7061 (1,7)	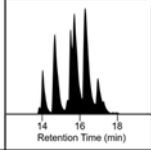	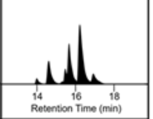
Iturins[2]	Antifungal	**C13** : $C_{47}H_{72}N_{12}O_{14}$<br>**C14** : $C_{48}H_{74}N_{12}O_{14}$<br>**C15** : $C_{49}H_{76}N_{12}O_{14}$<br>**C16** : $C_{50}H_{78}N_{12}O_{14}$<br>**C17** : $C_{51}H_{80}N_{12}O_{14}$	1043.5510 (1,0)<br>1057.5653 (2,2)<br>1071.5809 (2,3)<br>1085.5967 (2,1)	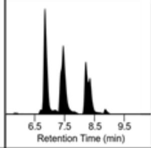	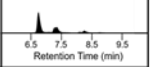
Fengycins[3]	Antifungal	**C15** : $C_{71}H_{108}N_{12}O_{20}$<br>**C16** : $C_{72}H_{110}N_{12}O_{20}$<br>**C17** : $C_{73}H_{112}N_{12}O_{20}$<br>**C18** : $C_{74}H_{114}N_{12}O_{20}$<br>**C19** : $C_{75}H_{116}N_{12}O_{20}$	1449.7806 (4,8)<br>1463.8004 (1,9)<br>1477.8171 (2,5)<br>1491.8305 (2,7)<br>1505.8440 (4,1)	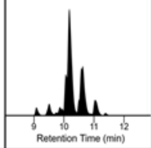	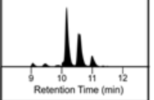
Difficidins[4]	Antibacterial	**Difficidin** : $C_{31}H_{45}O_6P$<br>**Oxy-** : $C_{31}H_{45}O_7P$	567.2828 (3,2)<br>583.2795 (7,8)	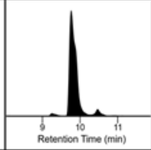	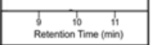
Bacillaenes[5]	Antibacterial	**Bacillaene** : $C_{34}H_{48}N_2O_6$<br>**Dihydro** : $C_{34}H_{50}N_2O_6$	581.3585 (2,3)<br>583.3726 (2,7)	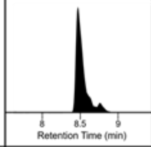	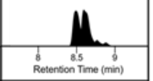
Macrolactins[6]	Antibacterial	**A** : $C_{24}H_{34}O_5$ (R=H)<br>**7-O-malonyl-A** : $C_{27}H_{36}O_8$ (R=CO-CH$_2$-COOH)<br>**7-O-succinyl-A** :$C_{28}H_{36}O_8$ (R=CO-CH$_2$-CH$_2$-COOH)<br>**D** : $C_{34}H_{48}O_{13}$ (R=6-O-syccinyl-β-gluc)	435.2298 (6,7)<br>511.2292 (2,0)<br>525.2459 (0,0)<br>687.2991 (0,6)	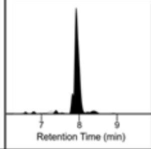	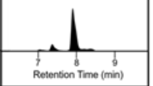
Bacillibactins[7]	Siderophore	$C_{39}H_{42}N_6O_{18}$	883.2620 (0,1)	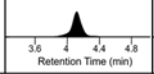	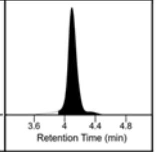

Figure S1. Diversity of predicted and detectable bioactive secondary metabolites (BSMs) produced by *B. velezensis* strain GA1. (A) Prediction of *B. velezensis* GA1 secondary metabolites. Biosynthetic gene clusters (genome annotation, Genbank: CP046386) were predicted by antiSMASH 6.0 (21). **(B)** GA1 BSMs and their corresponding main described activities and raw formula. Detected m/z ($[M+H]^+$) of each variant and mass error (in ppm, compared to theoretical mass) are calculated based on UPLC-MS analyses of supernatants coming from exudate mimicking (EM) and casamino acid (CAA) medium culture. Relative abundance of each BSMs is shown for both media. The numbers (superscript) next to the molecule name indicate the corresponding BSMs structure depicted in **C**. Structural changes leading to variants are indicated in red.

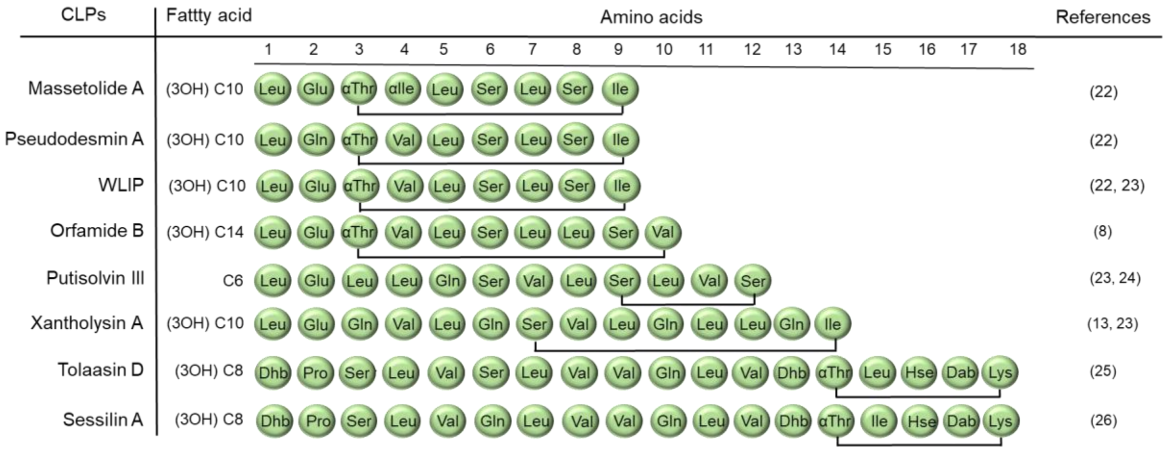

Figure S2. Simplified structural representation of the cyclic lipopeptides (CLPs) produced by *Pseudomonas* strains used in this study. The fatty acid chain length is presented and the amino acid composition are depicted indicating where cyclization occurs. For each CLP, only the main form detected is represented, for more details about minor variants see the also corresponding associated references (8, 13, 22–26).

A

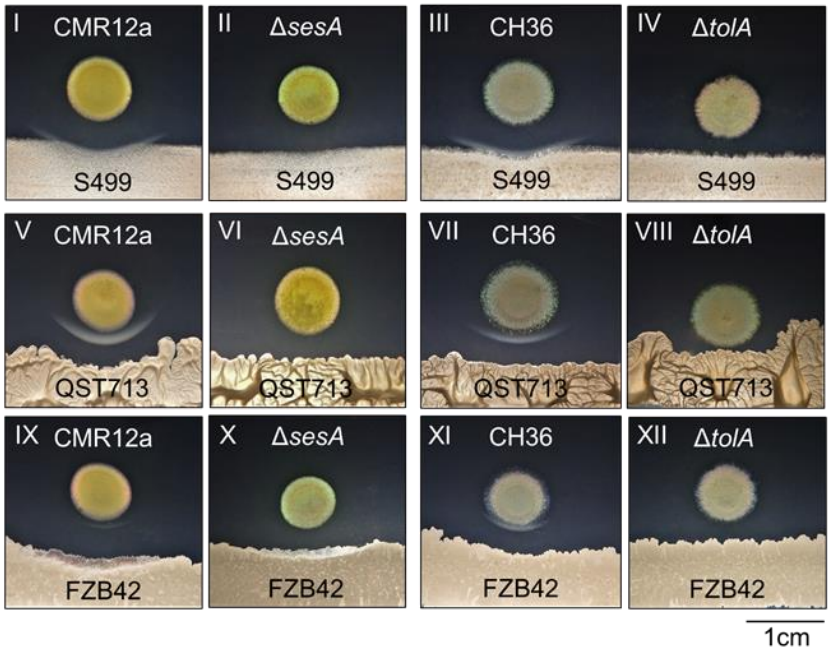

I CMR12a
II ΔsesA
III CH36
IV ΔtolA
S499
S499
S499
S499
V CMR12a
VI ΔsesA
VII CH36
VIII ΔtolA
QST713
QST713
QST713
QST713
IX CMR12a
X ΔsesA
XI CH36
XII ΔtolA
FZB42
FZB42
FZB42
FZB42
1cm

B

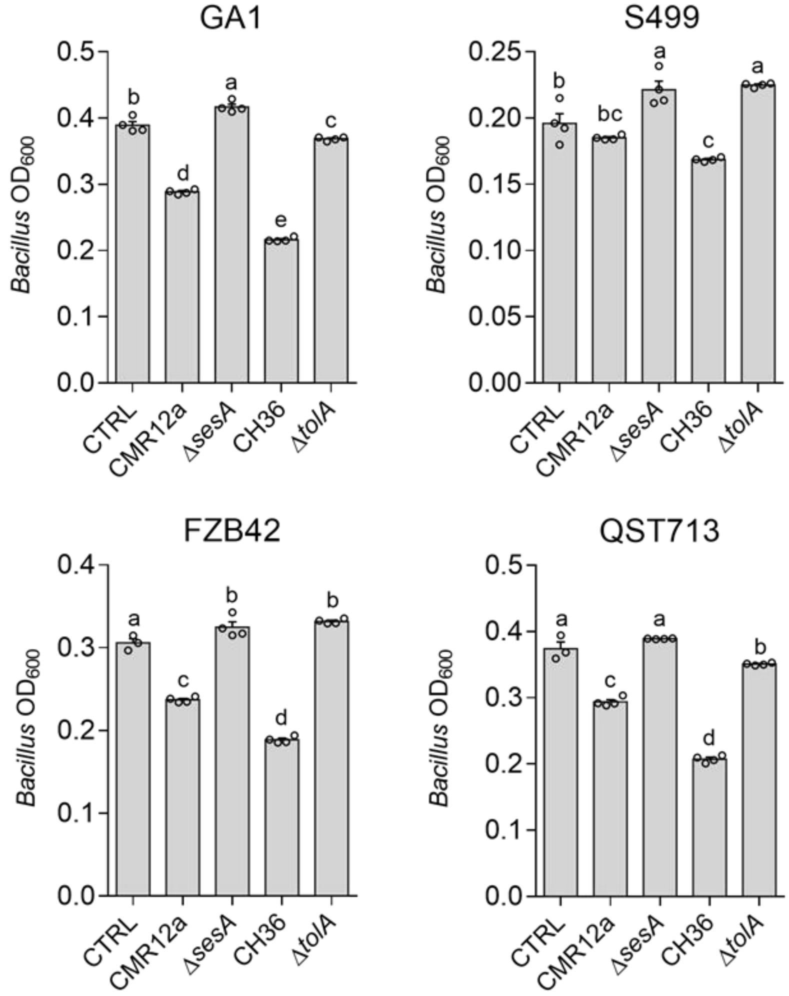

GA1
S499
FZB42
QST713
Bacillus OD600
CTRL
CMR12a
ΔsesA
CH36
ΔtolA
b
d
a
e
c
bc
a
c
a
a
c
b
d
b
a
c
a
d
b

Figure S3. The interplay between surfactins and sessilins/tolaasins is conserved within *B. velezensis.* (A) The white-line formation is dependent on the co-presence of surfactins and sessilins/tolaasins producers while during the interaction between *B. velezensis* wild-type strains (S499, QST713, FZB42) and *P. sessilinigenes* CMR12a (CMR12a) or *P. tolaasii* CH36 (CH36) mutants impaired in the production of sessilins (Δ*sesA*) or tolaasins (Δ*tolA*), respectively, no white line is observed. Sessilins/tolaasins production induces slight growth inhibition of all tested *B. velezensis* strains (panels I, III, V, VII, IX, XI). No inhibition is observed in confrontation assays with *Pseudomonas* mutants unable to produce sessilins or tolaasins (panels II, IV, VI, VIII, X, XII). The figure shows one representative repetition of three biological replicates with three technical repetitions (n=9). **(B)** Optical density (at 600nm) of different *B. velezensis* (GA1, S499, FZB42 and QST713) after 10 h culture supplemented with 4% (v/v) CMR12a or CH36 supernatants and their mutants unable to produce sessilins or tolaasins, respectively. Un-supplemented culture is represented as control (CTRL). Graphs show mean ± SD calculated from four replicate cultures (n = 4). Letters a to d indicate statistically significant differences according to Tukey's HSD test (α= 0.05).

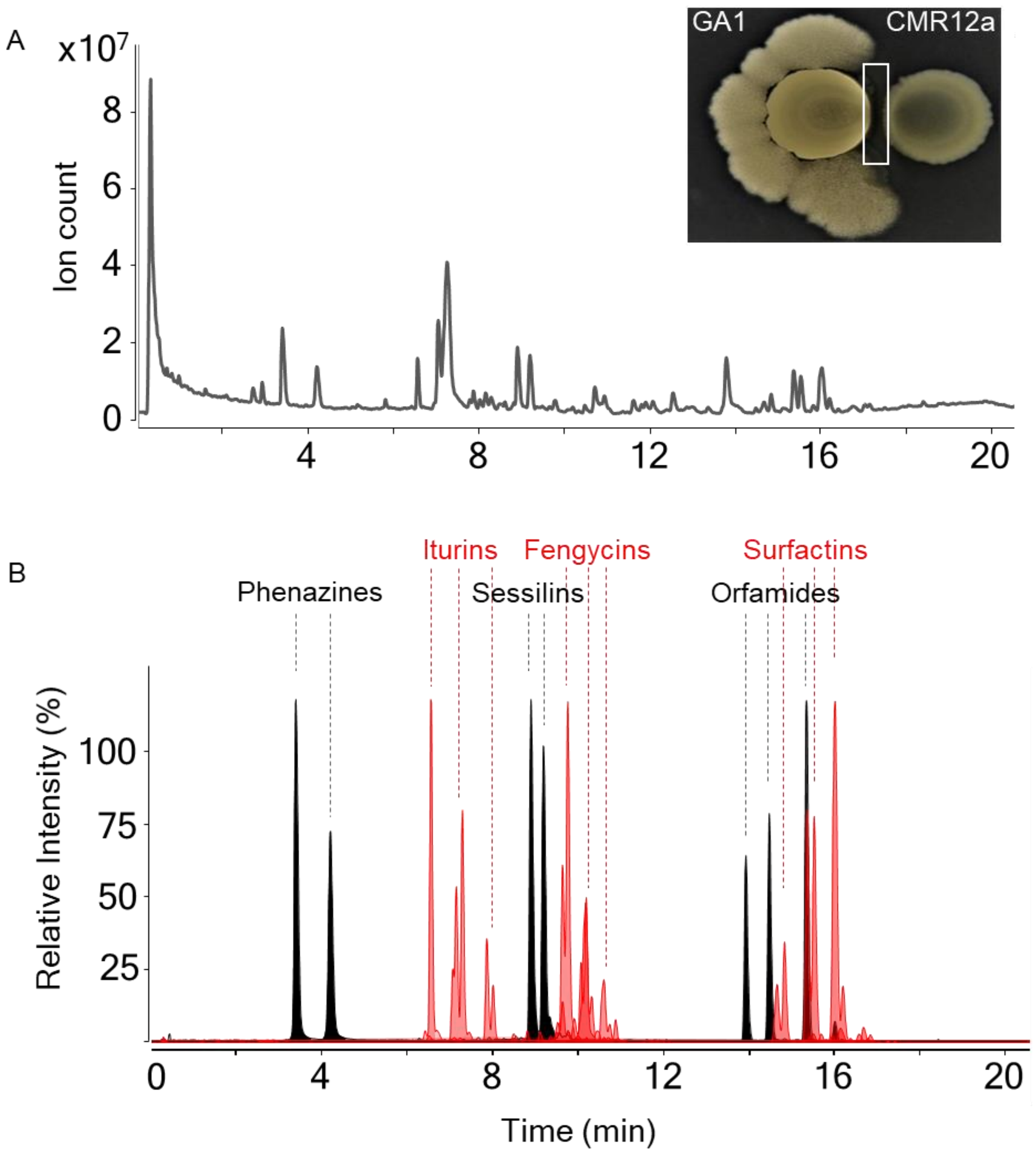

Figure S4. ***P. sessilinigenes*** **CMR12a and** ***B. velezensis*** **GA1 BSMs production upon confrontation on solid EM medium. (A)** Total ion chromatogram (TIC) and **(B)** Extracted ion chromatograms (EICs) overlaid for each BSMs produced by CMR12a (black) and GA1 (red) in the confrontation zone (labelled as a white rectangle on the upper-right confrontation figure) on solid EM medium after 24h. The intensity was fixed

to 100% for the main variant of each family. The figure shows one representative repetition of two biological replicates with three technical repetitions (n=6).

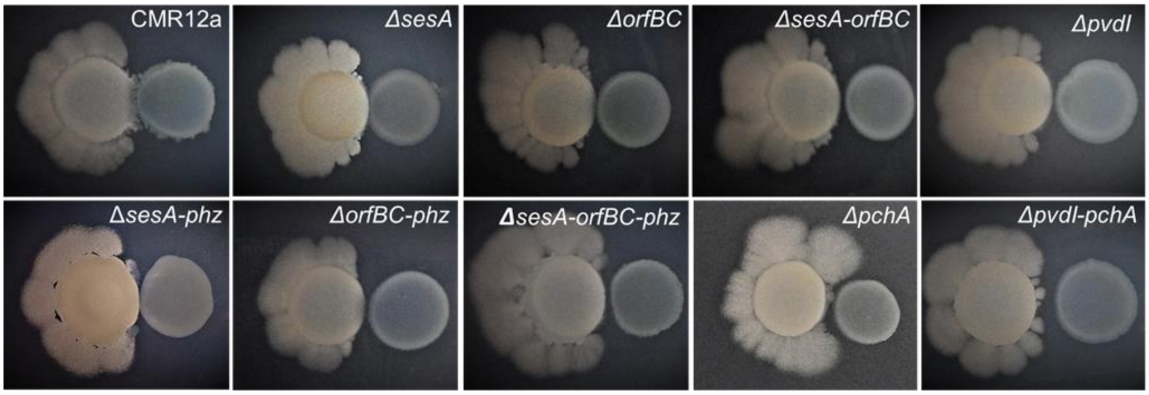

Figure S5: Effect of *P. sessilinigenes* CMR12a mutants on *B. velezensis* GA1 motility. GA1 (left colony) enhanced motility upon confrontation with CMR12a wild-type (CMR12a) or mutants repressed in the synthesis of sessilins (Δ*sesA*), orfamides (Δ*ofaBC*), orfamides and phenazines (Δ*ofaBC-phz*), sessilins and orfamides (*ΔsesA-ofaBC*), sessilins and phenazines (Δ*sesA-phz*), their triple mutant (Δ*sesA-ofaBC-phz*), pyoverdine (Δ*pvdI*), enantio-pyochelin (Δ*pchA*) or their double mutant (Δ*pvdI-pchA*) (see Table S1 for metabolome of each mutant). The figure shows one representative repetition of three biological replicates with three technical repetitions (n=9).

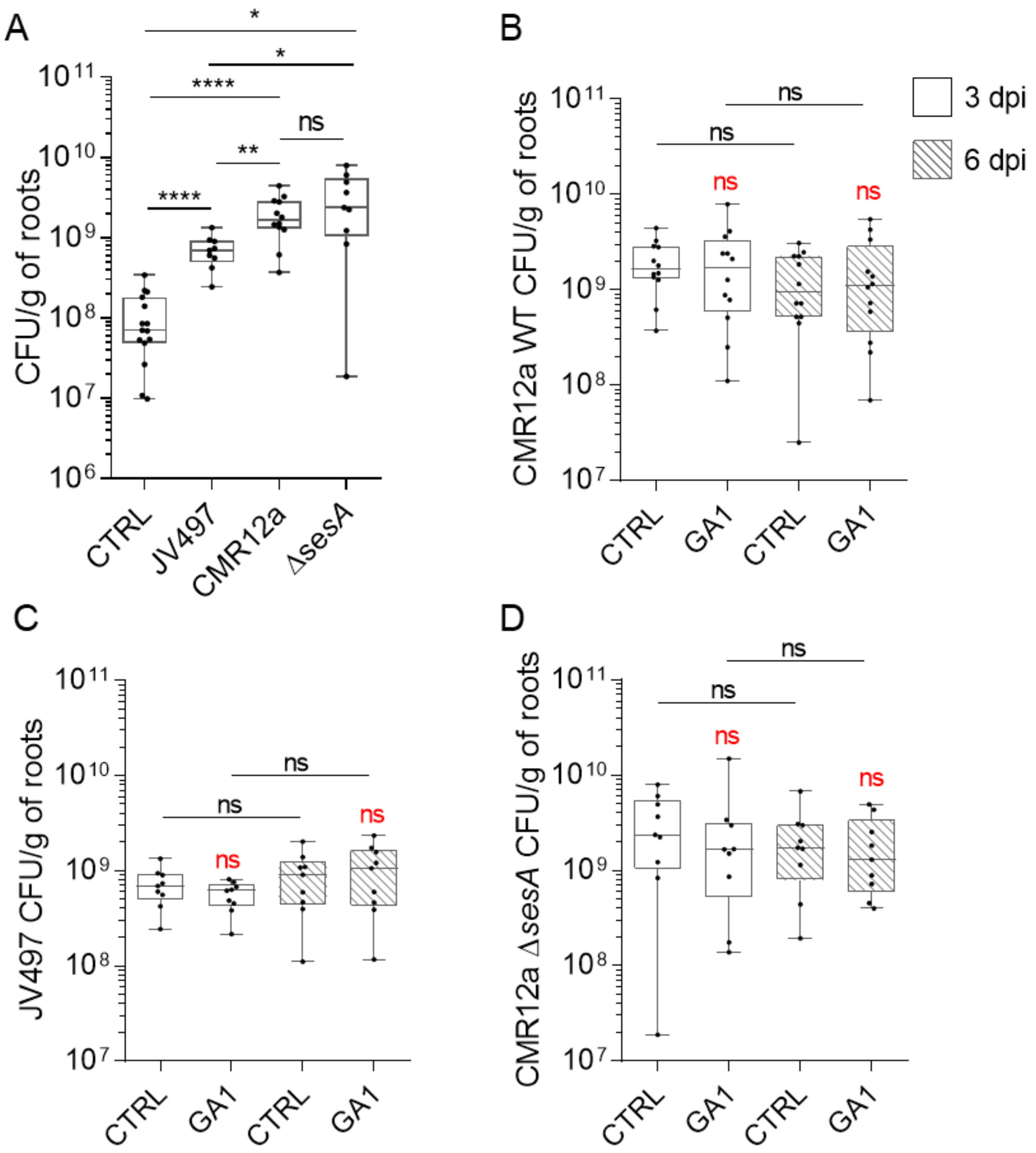

Figure S6. ***B. velezensis*** **GA1 and** ***Pseudomonas*** **spp. tomato root colonization.**

(A) GA1, JV497, CMR12a and Δ*sesA* population, expressed as colony forming units per gram of tomato roots (CFU/g of roots) recovered three days after single inoculation. Comparison between single (CTRL) and co-colonization (GA1) of tomato roots (CFU/g of roots) by CMR12a **(B),** JV497 **(C)**, and CMR12a mutant Δ*sesA* **(D)** between three (3

dpi, empty bars) and six (6 dpi, hatched bars) days after (co-)inoculation. Box plots were generated based on data from at least three independent assays each involving 6 plants per treatment (n=18). The whiskers extend to the minimum and maximum values, and the midline indicates the median. Statistical comparisons between treatments were realized with Mann–Whitney-test,’’ns’’ no significant difference and “****”, $P<0.0001$. The statistical differences between mono- and co-inoculated conditions are indicated as red-coloured labels while the statistical differences of the same condition between 3 and 6 dpi are indicated as black-coloured labels.

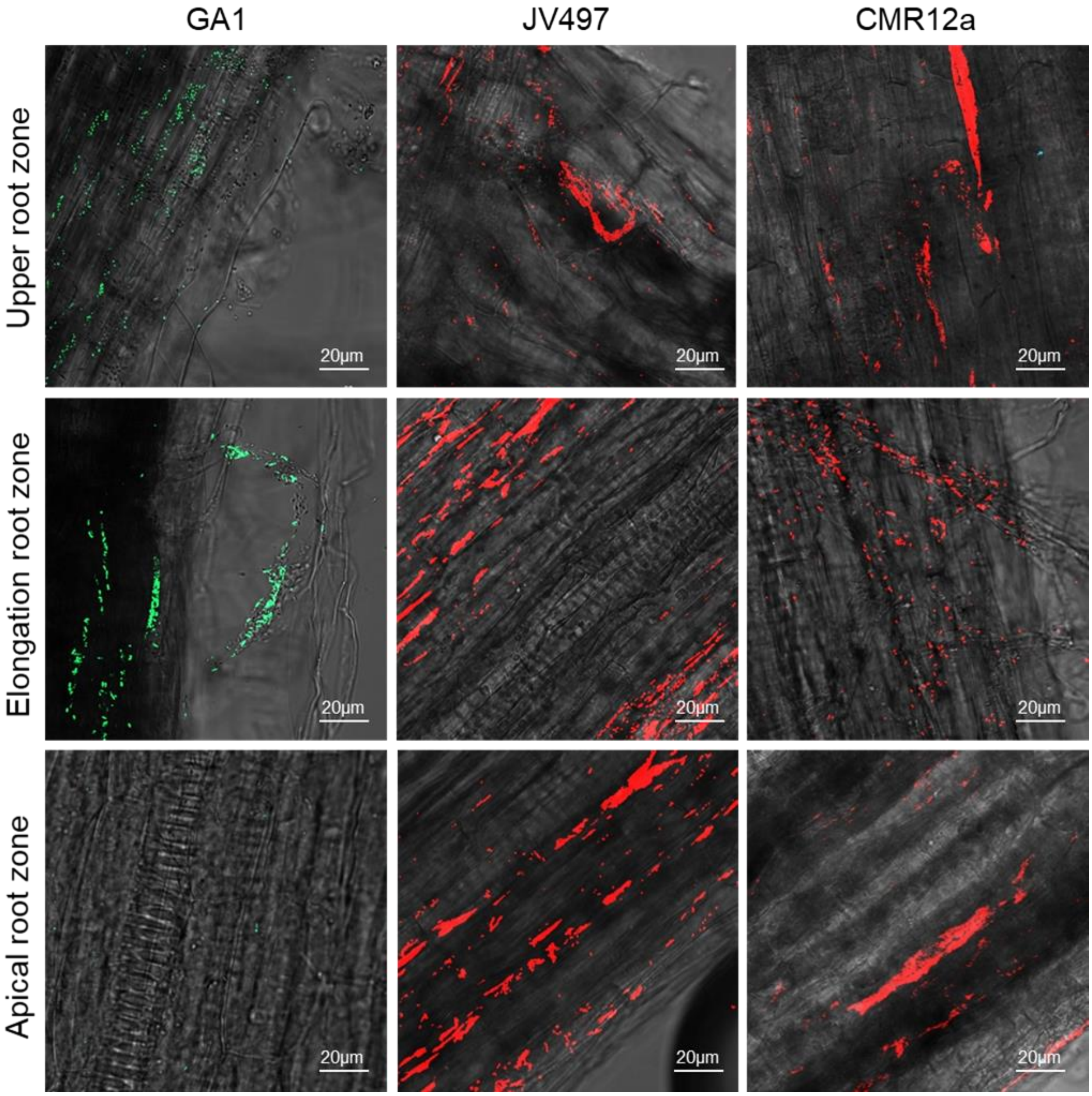

Figure S7. Confocal laser microscopy images of colonization and distribution of *B. velezensis* GA1, *P. chlororaphis* JV497 or *P. sessilinigenes* CMR12a along tomato roots. GA1, JV497, CMR12a root colonization and distribution on apical, elongation and upper root zones of tomato, 6 dpi. Cells expressing *GFPmut3* are green and correspond to GA1 cells while *mCherry*-labeled cells are red and correspond to

Pseudomonas spp. cells. The grey backgrounds correspond to root views observed with transmitted light. Images are representative of the analysis of 9 images per condition.

References

1. Touré Y, Ongena M, Jacques P, Guiro A, Thonart P. 2004. Role of lipopeptides produced by *Bacillus subtilis* GA1 in the reduction of grey mould disease caused by *Botrytis cinerea* on apple. *J Appl Microbiol* 96:1151–1160.

2. Molinatto G, Puopolo G, Sonego P, Moretto M, Engelen K, Viti C, Ongena M, Pertot I. 2016. Complete genome sequence of *Bacillus amyloliquefaciens* subsp. *plantarum* S499, a rhizobacterium that triggers plant defences and inhibits fungal phytopathogens. J Biotechnol 238:56–59.

3. Chen XH, Koumoutsi A, Scholz R, Eisenreich A, Schneider K, Heinemeyer I, Morgenstern B, Voss B, Hess WR, Reva O, Junge H, Voigt B, Jungblut PR, Vater J, Süssmuth R, Liesegang H, Strittmatter A, Gottschalk G, Borriss R, Sussmuth R, Liesegang H, Strittmatter A, Gottschalk G, Borriss R, Süssmuth R, Liesegang H, Strittmatter A, Gottschalk G, Borriss R, Sussmuth R, Liesegang H, Strittmatter A, Gottschalk G, Borriss R. 2007. Comparative analysis of the complete genome sequence of the plant growth-promoting bacterium *Bacillus amyloliquefaciens* FZB42. Nat Biotechnol 25:1007–1014.

4. Pandin C, Le Coq D, Deschamps J, Védie R, Rousseau T, Aymerich S, Briandet R. 2018. Complete genome sequence of *Bacillus velezensis* QST713: A

biocontrol agent that protects *Agaricus bisporus* crops against the green mould disease. J Biotechnol 278:10–19.

5. Perneel M, Heyrman J, Adiobo A, De Maeyer K, Raaijmakers JM, De Vos P, Höfte M. 2007. Characterization of CMR5c and CMR12a, novel fluorescent *Pseudomonas* strains from the cocoyam rhizosphere with biocontrol activity. *J* Appl Microbiol 103:1007–1020.

6. Girard L, Lood C, Höfte M, Vandamme P, Rokni-Zadeh H, Van Noort V, Lavigne R, De Mot R. 2021. The ever-expanding *Pseudomonas* genus: Description of 43 new species and partition of the *Pseudomonas* putida group. Microorganisms 9:1766.

7. D'aes J, Hua GKH, De Maeyer K, Pannecoucque J, Forrez I, Ongena M, Dietrich LEP, Thomashow LS, Mavrodi D V., Höfte M. 2011. Biological control of rhizoctonia root rot on bean by phenazineand cyclic lipopeptide-producing *Pseudomonas* CMR12a. Phytopathology 101:996–1004.

8. D'aes J, Kieu NP, Léclère V, Tokarski C, Olorunleke FE, De Maeyer K, Jacques P, Höfte M, Ongena M. 2014. To settle or to move? The interplay between two classes of cyclic lipopeptides in the biocontrol strain *Pseudomonas* CMR12a. Environ Microbiol 16:2282–2300.

9. Paulsen IT, Press CM, Ravel J, Kobayashi DY, Myers GSA, Mavrodi D V., DeBoy RT, Seshadri R, Ren Q, Madupu R, Dodson RJ, Durkin AS, Brinkac LM, Daugherty SC, Sullivan SA, Rosovitz MJ, Gwinn ML, Zhou L, Schneider DJ,

Cartinhour SW, Nelson WC, Weidman J, Watkins K, Tran K, Khouri H, Pierson EA, Pierson LS, Thomashow LS, Loper JE. 2005. Complete genome sequence of the plant commensal *Pseudomonas fluorescens* Pf-5. Nat Biotechnol 23:873–878.

10. Jousset A, Schuldes J, Keel C, Maurhofer M, Daniel R, Scheu S, Thuermer A. 2014. Full-genome sequence of the plant growth-promoting bacterium *Pseudomonas protegens* CHA0. Genome Announc 2:322–336.

11. Rieusset L, Rey M, Muller D, Vacheron J, Gerin F, Dubost A, Comte G, Prigent-Combaret C. 2020. Secondary metabolites from plant-associated *Pseudomonas* are overproduced in biofilm. Microb Biotechnol 13:1562–1580.

12. Rokni-Zadeh H, Li W, Sanchez-Rodriguez A, Sinnaeve D, Rozenski J, Martins JC, De Mot R. 2012. Genetic and functional characterization of cyclic lipopeptide white-line-inducing principle (WLIP) production by rice rhizosphere isolate *Pseudomonas putida* RW10S2. Appl Environ Microbiol 78:4826–4834.

13. Li W, Rokni-Zadeh H, De Vleeschouwer M, Ghequire MGK, Sinnaeve D, Xie GL, Rozenski J, Madder A, Martins JC, De Mot R. 2013. The antimicrobial compound xantholysin defines a new group of *Pseudomonas* cyclic lipopeptides. PLoS One 8:e62946.

14. Oni FE, Geudens N, Omoboye OO, Bertier L, Hua HGK, Adiobo A, Sinnaeve D, Martins JC, Höfte M. 2019. Fluorescent *Pseudomonas* and cyclic lipopeptide diversity in the rhizosphere of cocoyam (*Xanthosoma sagittifolium*). Environ Microbiol 21:1019–1034.

15. Loper JE, Hassan KA, Mavrodi D V., Davis EW, Lim CK, Shaffer BT, Elbourne LDH, Stockwell VO, Hartney SL, Breakwell K, Henkels MD, Tetu SG, Rangel LI, Kidarsa TA, Wilson NL, van de Mortel JE, Song C, Blumhagen R, Radune D, Hostetler JB, Brinkac LM, Durkin AS, Kluepfel DA, Wechter WP, Anderson AJ, Kim YC, Pierson LS, Pierson EA, Lindow SE, Kobayashi DY, Raaijmakers JM, Weller DM, Thomashow LS, Allen AE, Paulsen IT. 2012. Comparative genomics of plant-associated *Pseudomonas* spp.: Insights into diversity and inheritance of traits involved in multitrophic interactions. PLoS Genet 8:e1002784.

16. Miguel RN, Barret M, Morrisey JP, Germaine K, Francisco MG, Barahona E, Navazo A, María SC, Moynihan JA, Giddens SR, Coppoolse ER, Muriel C, Stiekema WJ, Rainey PB, Dowling D, O'gara F, Martín M, Rivilla R. 2012. Genome sequence of the biocontrol strain *Pseudomonas fluorescens* F113. J Bacteriol 194:1273–1274.

17. Hanahan D. 1983. Studies on transformation of *Escherichia coli* with plasmids. J Mol Biol 166:557–580.

18. Bisicchia P, Botella E, Devine KM. 2010. Plasmid Suite of novel vectors for ectopic insertion of GFP , CFP and IYFP transcriptional fusions in single copy at the *amyE* and *bglS* loci in *Bacillus subtilis*. Plasmid 64:143–149.

19. Meyer T, Vigouroux A, Aumont-Nicaise M, Comte G, Vial L, Lavire C, Moréra S. 2018. The plant defense signal galactinol is specifically used as a nutrient by the bacterial pathogen *Agrobacterium fabrum*. J Biol Chem 293:7930–7941.

20. Martínez-García E, de Lorenzo V. 2011. Engineering multiple genomic deletions in Gram-negative bacteria: analysis of the multi-resistant antibiotic profile of *Pseudomonas putida* KT2440. Environ Microbiol 13:2702–2716.

21. Blin K, Shaw S, Kloosterman AM, Charlop-Powers Z, Van Wezel GP, Medema MH, Weber T. 2021. antiSMASH 6.0: improving cluster detection and comparison capabilities. Nucleic Acids *Res* 49:W29–W35.

22. Sinnaeve D, Michaux C, Van hemel J, Vandenkerckhove J, Peys E, Borremans FAM, Sas B, Wouters J, Martins JC. 2009. Structure and X-ray conformation of pseudodesmins A and B, two new cyclic lipodepsipeptides from *Pseudomonas* bacteria. Tetrahedron 65:4173–4181.

23. Oni FE, Geudens N, Olorunleke OF, Salami AE, Omoboye OO, Arias AA, Adiobo A, Neve SD, Ongena M, Martins JC, Höfte M. 2020. Cyclic lipopeptide-producing *Pseudomonas koreensis* group strains dominate the cocoyam rhizosphere of a *Pythium* root rot suppressive soil contrasting with *P. putida* prominence in conducive soils. Env Microbiol 22:5137–5155.

24. Kuiper I, Lagendijk EL, Pickford R, Derrick JP, Lamers GEM, Thomas-Oates JE, Lugtenberg BJJ, Bloemberg G V. 2003. Characterization of two *Pseudomonas putida* lipopeptide biosurfactants, putisolvin I and II, which inhibit biofilm formation and break down existing biofilms. Mol Microbiol 51:97–113.

25. Bassarello C, Lazzaroni S, Bifulco G, Lo Cantore P, Iacobellis NS, Riccio R, Gomez-Paloma L, Evidente A. 2004. Tolaasins A−E, Five New Lipodepsipeptides

Produced by *Pseudomonas tolaasii*. J Nat Prod 67:811–816.

26. Ma Z, Hoang Hua GKH, Ongena M, Höfte M. 2016. Role of phenazines and cyclic lipopeptides produced by *Pseudomonas* sp. CMR12a in induced systemic resistance on rice and bean. Environ Microbiol Rep 8:896–904.
